# Quality evaluation of four *Ferula* plants and identification of their key volatiles based on non-targeted metabolomics

**DOI:** 10.3389/fpls.2023.1297449

**Published:** 2024-01-04

**Authors:** Meng Jiang, Mengwen Peng, Yuxia Li, Guifang Li, Xiaobo Li, Li Zhuang

**Affiliations:** ^1^ College of Life Sciences, Shihezi University, Shihezi, Xinjiang, China; ^2^ Xinjiang Production and Construction Corps Key Laboratory of Oasis Town and Mountain-basin System Ecology, Shihezi, Xinjiang, China; ^3^ Xinjiang Compass Biotechnology Co., Ltd, Changji, Xinjiang, China

**Keywords:** edible and medicinal plants (EMPs), genus *Ferula*, HS-SPME-GC-MS, volatile organic compounds (VOCs), bioactive component, non-targeted metabolomics

## Abstract

**Introduction:**

*Ferula* is a traditional, edible, and important medicinal plant with high economic value. The distinction between edible and non-edible *Ferula* remains unclear.

**Methods:**

In this study, headspace solid-phase microextraction coupled to gas chromatography-mass spectrometry (HS-SPME/GC-MS) and ultra-high performance liquid chromatography-tandem mass spectrometry (UHPLC-MS/MS) non-targeted metabolomics techniques were used to systematically and comprehensively analyse secondary metabolites in the leaves and roots of four species of *Ferula*, considering their edibility.

**Results:**

A total of 166 leaf volatile organic compounds (VOCs) and 1,079 root metabolites were identified. Additionally, 42 potential VOCs and 62 differential root metabolites were screened to distinguish between edible and non-edible *Ferula*. Twelve volatile metabolites were specific to *F. feurlaeoides*, and eight compounds were specific to the three edible *Ferula* species. The results showed that compounds containing sulphur, aldehydes, and ketones, which produce pungent odours, were the primary sources of the strong odour of *Ferula*. The root differential metabolites include 13 categories, among which the high concentration group is organic acids, amino acids, terpenoids and fatty acids. The bioactive metabolites and VOCs in the roots exhibited species-specific characteristics. VOCs with various odors were linked to the distribution of root metabolites in both edible and non-edible *Ferula* plants. The screened root markers may contribute to the formation of characteristic VOCs.

**Discussion:**

This study identified the difference in flavour between edible and non-edible *Ferula* plants and, for the first time, demonstrated the contribution of the efficacy of *Ferula* root to the unique flavour of the above-ground parts of *Ferula*. These results provide a theoretical basis for selecting *Ferula* for consumption and help evaluate the quality of different species of *Ferula*. Our findings may facilitate food processing and the further development of *Ferula*.

## Introduction

Pharmacologically active ingredients of medicinal plants have provided rich resources for the development of new drugs (Rahman et al., 2019). Several medicinal plants are also edible and can supply the necessary nutrients to the body; such plants are known as edible and medicinal plants (EMPs). EMPs contain high amounts of vitamins, fibre, and essential amino acids, as well as other medicinally active ingredients essential for good health, and some EMPs are more popular than others owing to their unique flavour. However, their nutritional and phytochemical compositions remain to be fully elucidated (Tan et al., 2022).

In recent years, interest in edible medicinal plants as new products with beneficial properties has increased (Cheng et al., 2022). Apiaceae is one of the most studied families of angiosperms with economic importance (Wei et al., 2019). Apiaceae plants are known for their production of aromatic compounds, particularly volatile oils, which contribute to their distinctive flavours and medicinal properties. These volatile oils are often rich in compounds such as terpenes, phenylpropanoids, and flavonoids, which have been extensively studied for their pharmacological activities (Amiri and Joharchi, 2016; Bahmankar et al., 2019; Mohammadhosseini et al., 2019; Akaberi et al., 2020). Furthermore, Apiaceae plants have significant ethnobotanical importance, being traditionally used by various ethnic groups for culinary, medicinal, and ritualistic purposes, and several species are consumed as wild vegetables or spices due to their high nutritional and medicinal value (Li et al., 2018). The Chinese government has promoted the cultivation and breeding of medicinal plants of the Apiaceae family, and various medicinal plants, such as *Panax ginseng* C.A. Meyer (He et al., 2022), *Angelica sinensis* (Oliv.) Diels (Xiao et al., 2021), and *Ligusticum chuanxiong* Hort (Chen et al., 2018), have been domesticated as cash crops.

*Ferula*, the third largest genus in the family Apiaceae, is distributed worldwide, particularly in Asia (Khayat et al., 2023) (**Supplementary Figure 1**). *Ferula* is a perennial herb that bears one or more fruits. Plants of this genus have been recorded in the Compendium of Materia Medica in China for 2000 years. The whole plant of most species has a potent smell like that of onions and garlic; its roots and rhizomes have been used as medicinal materials due to their immunosuppressive, antipyretic, analgesic, and anti-inflammatory activities (Amalraj and Gopi, 2017; Zhang et al., 2021). During early spring in Central Asia, the crops are not fully grown; however, *Ferula* leaves grow and are collected as a vegetable due to their unique flavour of these leaves. In addition, its dried roots or leaves are used as spices; therefore, the whole plant has important economic value and is an important EMP (Radulović et al., 2013). Owing to the special flavour of its leaves and the excellent medicinal value of the roots, *Ferula* has been used as a special food in China. For the protection of *Ferula* resources and their sustainable development, research and planting have been conducted in Xinjiang, the main *Ferula* production area in China.

Previous studies on *Ferula* have focused on the analysis of its chemicals and validation of their medicinal value (Mohammadhosseini et al., 2019); for instance, the natural extract of *Ferula* has been reported to exhibit antibacterial, anticancer, antioxidant and melanogenesis inhibitory effects (Charmforoshan et al., 2019; Kamelan Kafi et al., 2022). Although there is a wealth of information on the biological activity of *Ferula*, few studies have simultaneously compared multiple types of *Ferula* species simultaneously. In addition, many of the parts used to study the biological activities were underground parts; therefore, little consideration has been given to the botanical characteristics of the aboveground parts, which comprise more organs. The unique odour of *Ferula* is attributed to the VOCs in the leaves, and research has shown that the VOCs of plants are not only aromatic components, but also exhibit various medicinal properties as natural compounds, including antibacterial effects (Ricciardi et al., 2021). Studies have also shown that VOCs are essential for defence responses and interactions between plants and microbes (Boncan et al., 2020; Feng et al., 2023). However, systematic studies on the overall VOC composition of *Ferula* plants are currently lacking. In addition, compared with the fast and high-throughput characteristics of omics, traditional nutrient composition detection methods only focus on the detection of a few compounds or a certain class of compounds, and cannot effectively reflect the whole picture of metabolic compounds in *Ferula*. Metabolomics, as a rapidly advancing omics technique, focuses on the comprehensive analysis and quantification of metabolites in distinct anatomical regions of higher plants. Its application extends to the investigation of medicinal plants and its relevance in the food industry has also been extensively explored (Mei et al., 2022; Utpott et al., 2022). Exploring the scientific connotations of traditional medicinal knowledge using phytochemistry, pharmacology, and metabolomics is the mainstream research direction for medicinal botany (Wang et al., 2023).

The unique odour of *Ferula* can help distinguish between edible and nonedible *Ferula* species. The more pungent the smell is, the better the medicinal value and the greater the benefits to the body; however, evidence to support this is lacking, and the correlation between the unique smell of *Ferula* and its medicinal value has not been fully studied. Specifically, two major questions remain to be answered: (1) can the smell be used to distinguish between edible and non-edible *Ferula* species? (2) what is the relationship between the smell and the medicinal components? To answer these questions, the compound compositions of the main medicinal part (root) and main edible part (leaf) of four species of *Ferula* were analysed in this study. Changes in the VOCs of different species of *Ferula* were assessed using headspace solid-phase microextraction coupled to gas chromatography-mass spectrometry (HS-SPME/GC-MS), while the metabolic profiles of different species of *Ferula* were investigated using the non-targeted metabolomics method, ultra-high performance liquid chromatography-tandem mass spectrometry (UHPLC-MS/MS). Overall, this study aimed to determine the metabolic characteristics of four species of *Ferula*, identify specific markers of the leaves and roots of *Ferula*, and elucidate the effects of roots on leaf metabolism. The results of this study will help clarify the contribution of VOCs to the leaf flavour, elucidate the effects of roots on leaf metabolism, and highlight the correlation between the effects of metabolism in the root and leaf.

## Materials and methods

### Plant material and sample collection

Leaves and roots of four species of *Ferula* were collected from farms in Shihezi City (Collection details are shown in **Supplementary Table 1**). The four species were identified as *Ferula lehmannii* Boiss. Fl. Or., *F. sinkiangensis* K. M. Shen., *F. teterrima* Kar.et Kir., and *F. feurlaeoides* (Steud.) Korov by Professor Yan Ping from the Herbarium of Shihezi University (SHI). Among them, *F. lehmannii*, *F. sinkiangensis*, and *F. teterrima* are medicinal plants with a strong odour throughout the plant and are commonly consumed as wild vegetables. *Ferula feurlaeoides* has a bitter smell and is not used as a vegetable or medicinal plant.

The collected leaf samples were immediately placed in liquid nitrogen and transported to the laboratory. All samples were stored at −80°C until analysis. After the fresh roots were collected, they were dried indoors and protected from direct sunlight. Three samples were collected for each species, and subsequent analysis was conducted after thorough drying.

### Reagents and standards

LC-MS grade methanol (MeOH) and LC-MS grade acetonitrile (ACN) were obtained from Fisher Scientific (Loughborough, UK). 2- Amino-3-(2-chloro-phenyl)-propionic acid and ammonium formate were obtained from Aladdin Company (Shanghai, China); while formic acid was sourced from TCI (Shanghai, China). The water was purified using the Millipore system (Millipore, Bedford).

### HS-SPME/GC-MS analysis of leaves

The determination was performed using the HS-SPME method. 1g leaves was placed in a 20mL headspace bottle, and then sealed with a rubber spacer-equipped bottle cap. The extraction was carried out using CTC Autosampler Syringes, extraction fibres: 50/30um DVB/CAR on PDMS (57348-U, Supelco, USA); Temperature: 50 °; He oscillation was conducted for 15 minutes followed by a 30-minute extraction period; Oscillation speed: 250 rpm; Analysis time: 5 min; GC cycle time: 50 min. After extraction, the probe was removed and inserted into the gas chromatography inlet. The extraction layer was then pushed out, and GC-MS was employed for the detection and analysis of volatile compounds.

GC conditions: The volatile matter was detected on an Agilent 7890 B gas chromatograph (Agilent Technologies, USA) coupled with an Agilent 5977B triple quadrupole mass spectrometry detector. DB-wax capillary column (30 m ×0.25 mm inner diameter ×0.25 μm film thickness) for the separation of volatile compounds. The initial column temperature was set at 40°C and held for 5 min. Then, the temperature was increased at a rate of 5°C/min until it reached 220°C. Subsequently, the temperature was further increased at a rate of 20/min to 250°C and held for 2.5 min. The injection temperature is 270°C and the shunt free mode is used with an inlet lining with an internal diameter of 0.75 mm. The flow rate of the carrier gas was set at 1.0 ml/min (99.999%).

MS condition: The ion source was operated in the EI+ mode with an electron ionization energy 70eV. The ion source temperature was set at 230 °, and the quadrupole temperature was set at 150 °. Full scan mode was used for mass spectrometry analysis with a scanning range of 20-400 *m/z*.

### Sample preparation and measurement for non-targeted metabolomics of roots

The sample was weighed and placed in a 2 mL centrifuge tube. Methanol (600 μL) containing 2-Amino-3-(2-chloro-phenyl)-propionic acid (4 ppm) was added and vortexed for 30 seconds. Glass beads (100 mg) were added and the mixture was ground for 90 seconds. Ultrasound treatment was conducted for 15 minutes at room temperature. After centrifugation at 12,000 rpm and 4°C for 10 minutes, the supernatant was filtered through a 0.22 μm membrane and transferred for subsequent analysis (Vasilev et al., 2016).

UHPLC-MS/MS analysis was performed on the Thermo Orbitrap Exploris 120 mass spectrometer (Thermo Fisher Scientific, USA) equipped with an Electrospray ion Source (ESI) and a Thermo Vanquish ultra-high performance Liquid chromatography (UHPLC) system (Thermo Fisher Scientific, USA). Chromatographic separation was conducted using ACQUITY UPLC^®^ HSS T3(2.1×150 mm, 1.8 μm) (Waters, Milford, MA, USA) column.

The automatic sampler and column temperatures were set at 10°C and 40°C, respectively. The flow rate in gradient mode was 0.25 mL/min. In the positive ion mode, the mobile phase consisted of formic acid water (0.1% formic acid aqueous solution, v/v) (mobile phase A) and acidified acetonitrile (0.1% acetonitrile solution, v/v) (mobile phase B). The linear gradient of mobile phase B was 0-1 min, 2% B; 1-9 min, 2%-50% B; 9-12 min, 50%-98% B; 12-13.5 min, 98% B; 13.5-14 min, 98%-2% B; 14-20 min, 2% B. In the negative ion mode, the mobile phase consisted of acetonitrile (C) and 5 mM ammonium formate water (D). The gradient elution procedure was as follows 0-1 min, 2% C; 1-9 min, 2%-50% C; 9-12 min, 50%-98% C; 12-13.5 min, 98% C; 13.5-14 min, 98%-2% C; 14-17 min, 2% VC (Zelena et al., 2009; Want et al., 2013).

A sample volume of 2 uL was injected into the system and analysed in positive and negative ion ionization (ESI) mode. The positive ion spray voltage was set at 3.50 kV, and the negative ion spray voltage was set at -2.50 kV. The capillary temperature was maintained at 325 °. For the first-level analysis, a full scan was performed at a resolution of 60,000, covering a mass-to-charge ratio (*m/z*)range of100-1000, and the second-level fragmentation was carried out using higher-energy collisional dissociation (HCD). with a collision voltage of 30%, the second-level analysis was performed at a resolution of 15,000, and the first four ions were subjected to fragmentation before signal collection. Additionally, dynamic exclusion was employed to eliminate redundant MS/MS information.

### Metabolome data preprocessing and multivariate statistical analysis

The identification of volatile compounds from HS-SPME/GC-MS data was performed based on their mass spectrum fragments. The identification process involved manual analysis and comparison with the National Institute of Standards and Technology (NIST Mass Spectral Library 2014). In addition, the mass nucleus ratio of the isolated volatile compounds was compared with the literature values to identify the GC-MS detection results, and the peak area normalization method was used to calculate the volatile compounds content.

The UHPLC-MS/MS data were converted into mzXML file format using the MSConvert tool in the Proteowizard software package (v3.0.8789). The quantitative list of substances was obtained by employing the R XCMS software package for peak detection, peak filtering and peak alignment, the parameters are bw = 2, ppm = 15, peakwidth = c (5, 30), mzwid = 0.015, mzdiff = 0.01, method=‘centWave’. Substance identification utilized public databases, including HMDB (http://www.hmdb.ca), massbank (http://www.massbank.jp/), LipidMaps (http://www.lipidmaps.org), mzcloud (https://www.mzcloud.org), KEGG (http://www.genome.jp/kegg/) and self-built material bank were used for substance identification, and the parameter with the parameter set to ppm < 30 ppm. The LOESS signal correction method based on QC sample, was employed for data correction and eliminates system error. After normalization, only ion peaks with relative standard deviations (RSDs) less than 30% in QC were kept to ensure proper metabolite identification. After the pretreatment of data had been submitted to the MetaboAnalyst (https://www.metaboanalyst.ca), used for principal component analysis (PCA), orthogonal partial least squares discriminant analysis (OPLS-DA)were performed. Clustering heatmap was performed based on the normalized data of the independent variables. Meanwhile, clustering heatmap analysis (HCA) was conducted using MetaboAnalyst 5.0. Screening criteria for volatile differential metabolites consisted of Fold Change ≥ 2 or Fold Change ≤ -2 and *p* < 0.05. The differential metabolites of the roots were screened by combining variables important in the projection variables important in the projection (VIP) > 1.0 and *p* < 0.05.The selected differential metabolites were enriched and analyzed according to metabolic pathways using MetaboAnalyst database. Variance analysis (ANOVA), significance analysis (*p* < 0.05), and mapping were performed using SPSS 19.0 (SPSS Inc., Chicago, IL, USA) and Origin 2023b software, respectively.

## Results

### Composition analysis of volatile metabolites in the leaves of four species of *Ferula*

**Figure 1:**
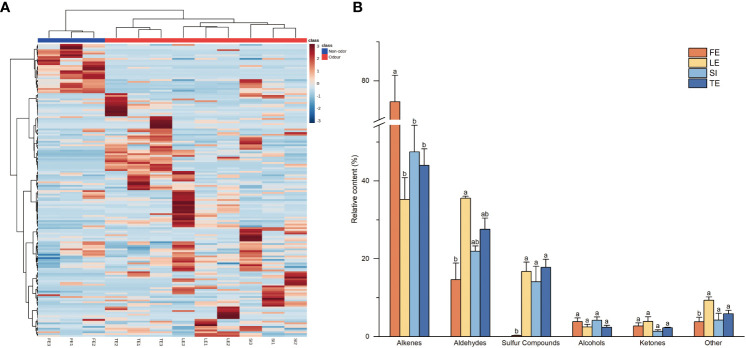
Heatmaps and type of measured volatiles in *Ferula* samples. **(A)** Heatmap of volatile metabolites. **(B)** Histogram of relative content of volatile metabolite components. FE, *F*. *feurlaeoide*; LE, *F. lehmannii*; SI, *F. sinkiangensis*; TE, *F*. *teterrima*. Different letters indicate significant species differences at the 0.05 level.

We assessed the volatile metabolites in leaf samples of *Ferula* and identified 166 VOCs (**Supplementary Table 2**), including 54 alkenes (Among them are 24 monoterpene hydrocarbons and 29 sesquiterpene hydrocarbons), 29 aldehydes, 21 ketones, 14 alcohols, 14 esters, 12 sulphur-containing compounds, 9 acids and 13 other compounds. Leaf flavour quality was affected by the content and proportion of aroma components. The clustering heat map (**Figure 1A**) showed that the leaves had abundant aroma components, all of which showed good repeatability within the group, and edible *Ferula* was well distinguished from nonedible *Ferula*. There were significant differences in the composition, content, proportion, and unique components, indicating obvious differences in aroma characteristics among the different species.

There were also significant differences in the contents of various compounds among the different species. As shown in **Figure 1B**, alkenes, aldehydes, and sulphur-containing compounds had the highest contents among the volatile compounds. Furthermore, alkene compounds were the most abundant (56). *Ferula feurlaeoides* contained significantly higher alkene compounds than the other species, while *F. lehmannii* contained 35.27% of the total alkene compounds identified.

Aldehydes were the second most abundant component (30); the content of aldehydes in *F. feurlaeoides* was significantly lower than that in the remaining species. Alcohols and ketones were less abundant than alkenes, aldehydes, and sulphur compounds, with no significant differences among the species. Although there were only 11 sulphur-containing compounds, the content was very high in the volatiles, and the content in *F. feurlaeoides* was very small (0.32%) in the four species of *Ferula*; this is consistent with the smell of the four species of *Ferula*. Other compounds, such as esters and acids, were present in relatively small amounts. Compounds with higher contents may have a higher contribution to the unique aroma of different flavours. The odour characteristics of 31 compounds with a high content (≥1%) in the four *Ferula* species are listed in **Table 1**. Among them, (*Z*)-sec-butyl propenyl disulfide, (*E*)-sec-butyl propenyl disulphide, and β-myrcene are the typical aroma components reported in *Ferula*.

**Table 1:** Odor characteristics of relatively high contents (≥1%) in *Ferula* samples.

Compound name	Class	Odor Type	Odor Description	Compound content/%			
				TE	LE	SI	FE
1-Penten-3-ol	Alcohols	green	ethereal, horseradish, vegetable	0.61 ± 0.59 a	0.04 ± 0.02 a	1.11 ± 1.04 a	1.32 ± 0.4 a
2-Penten-1-ol, (Z)-	Alcohols	green	phenolic, nasturtium, medicinal	1.75 ± 0.29 ab	2.4 ± 0.69 ab	2.78 ± 0.63 a	0.98 ± 0.28 b
1-Hexanol	Alcohols	herbal	ethereal, fusel, oil, alcoholic	0 ± 0 b	0 ± 0 b	0 ± 0 b	1.27 ± 0.62 a
Acetaldehyde, hydroxy-	Aldehydes	–	–	0.93 ± 0.04 b	1.51 ± 0.07 b	2.83 ± 0.17 a	0.96 ± 0.3 b
2,4-Hexadienal, (E,E)-	Aldehydes	green	sweet, green, spicy, floral	1.87 ± 0.93 a	1.94 ± 1.61 a	0.62 ± 0.57 a	0.2 ± 0.19 a
2-Hexenal	Aldehydes	green	sweet, almond, bitter, almon	16.9 ± 2.72 a	14.01 ± 6.29 a	14.87 ± 9.05 a	7.76 ± 2.44 a
Hexanal	Aldehydes	green	fresh, green, fatty, aldehydic	5.75 ± 0.61 b	14.15 ± 3.69 a	0.04 ± 0 b	2.02 ± 0.9 b
2,4-Heptadienal, (E,E)-	Aldehydes	fatty	fatty, green, oily, aldehydic	0.97 ± 0.13 a	1.43 ± 0.3 a	1.49 ± 0.34 a	1.15 ± 0.56 a
o-Cymene	monoterpene hydrocarbons	floral	–	0.68 ± 0.02 c	0.4 ± 0.2 c	17.67 ± 3 b	32.3 ± 1.99 a
(1R)-2,6,6-Trimethylbicyclo[3.1.1]hept-2-ene	monoterpene hydrocarbons	herbal	harsh, terpene, aromatic, minty	1.79 ± 0.21 a	1.12 ± 0.48 a	1.08 ± 0.69 a	2.44 ± 0.52 a
β-pinene	monoterpene hydrocarbons	herbal	dry, woody, fresh, pine, green	13.12 ± 2.35 a	7.73 ± 2.8 ab	3.52 ± 0.4 bc	0.76 ± 0.36 c
Sabinene	monoterpene hydrocarbons	woody	woody, spicy, camphoreous	1.75 ± 0.24 a	2.26 ± 0.9 a	1.39 ± 0.75 a	10.38 ± 5.86 a
α-phellandrene	monoterpene hydrocarbons	terpenic	citrus, herbal, terpenic, green	0 ± 0 b	0.23 ± 0.14 b	0 ± 0 b	2.96 ± 1.51 a
β-Myrcene	monoterpene hydrocarbons	spicy	terpy, herbaceous, woody	0.11 ± 0.09 c	0.63 ± 0.3 c	4.66 ± 1.46 b	12.73 ± 1.77 a
D-Limonene	monoterpene hydrocarbons	citrus	citrus, orange, fresh, sweet	9.56 ± 2.5 a	11.19 ± 5.92 a	4.85 ± 2.81 a	5.5 ± 4.07 a
γ-Terpinene	monoterpene hydrocarbons	terpenic	terpy, sweet, citrus,tropical	0.04 ± 0.01 a	0.83 ± 0.54 a	9.51 ± 4.86 a	6.28 ± 6.09 a
Terpinolene	monoterpene hydrocarbons	herbal	fresh, woody, sweet, pine,	4.37 ± 0.99 a	0 ± 0 b	0 ± 0 b	0.05 ± 0.05 b
Furan, 3-(4-methyl-3-pentenyl)-	sesquiterpene hydrocarbons	woody	–	0.24 ± 0.01 b	2.14 ± 0.96 a	0.16 ± 0.12 b	0 ± 0 b
(*E*)-4,8-Dimethylnona-1,3,7-triene	sesquiterpene hydrocarbons	–	–	0.45 ± 0.05 b	2.68 ± 1.34 a	0.23 ± 0.11 b	0 ± 0 b
Copaene	sesquiterpene hydrocarbons	woody	woody, spicy, honey	1.7 ± 1.13 a	0.18 ± 0.1 a	0.35 ± 0.29 a	0 ± 0 a
α-Cubebene	sesquiterpene hydrocarbons	herbal	herbal, waxy	1.1 ± 0.58 a	0.53 ± 0.16 bc	0.26 ± 0.14 bc	0 ± 0 b
Germacrene D	sesquiterpene hydrocarbons	woody	woody, spicy	2.06 ± 1.01 a	0.08 ± 0.04 b	0.57 ± 0.3 ab	0 ± 0 b
*n*-Caproic acid vinyl ester	Esters	–	–	0.83 ± 0.52 ab	2.3 ± 0.75 a	1.14 ± 0.36 ab	0.22 ± 0.13 b
1-Penten-3-one	Ketones	spicy	pungent, peppery, garlic, onion	1.7 ± 0.01 ab	2.08 ± 0.63 a	0.61 ± 0.55 bc	0 ± 0 c
5-Hepten-2-one, 6-methyl-	Ketones	citrus	citrus, orange, fresh, sweet	0.03 ± 0.01 b	0.03 ± 0.01 b	0.06 ± 0.01 b	1.88 ± 0.69 a
Furan, 2-ethyl-	Other	chemical	beany, ethereal, cocoa, bready	2.66 ± 0.46 a	4.24 ± 1.88 a	1.12 ± 0.98 a	1.44 ± 0.6 a
Carbamic acid, monoammonium salt	Other	–	–	0.72 ± 0.28 a	0.99 ± 0.39 a	0.84 ± 0.35 a	1.08 ± 0.37 a
(*Z*)-sec-Butyl propenyl disulfide	Sulfurous compound	Sulfurous	–	1.06 ± 1.06 a	0.04 ± 0.02 a	2.75 ± 1.38 a	0 ± 0 a
(*E*)-sec-Butyl propenyl disulfide	Sulfurous compound	Sulfurous	–	2.6 ± 1.59 a	0.14 ± 0.13 a	1.62 ± 0.8 a	0 ± 0 a
1-(1-(Methylthio) propyl)-2-propyldisulfane	Sulfurous compound	sulfurous	–	13.41 ± 4 a	7.37 ± 3.37 a	11.79 ± 8.78 a	0.2 ± 0.09 a
Disulfide, methyl 1-(methylthio) propyl	Sulfurous compound	Sulfurous	–	0.02 ± 0.02 a	5.84 ± 3.12 a	3.86 ± 3.84 a	0.11 ± 0.06 a

Mean ± SE, n = 3. Different letters indicate significant species differences at the 0.05 level.

### Screening of different VOCs from edible and non-edible *Ferula* with different flavours

**Figure 2:**
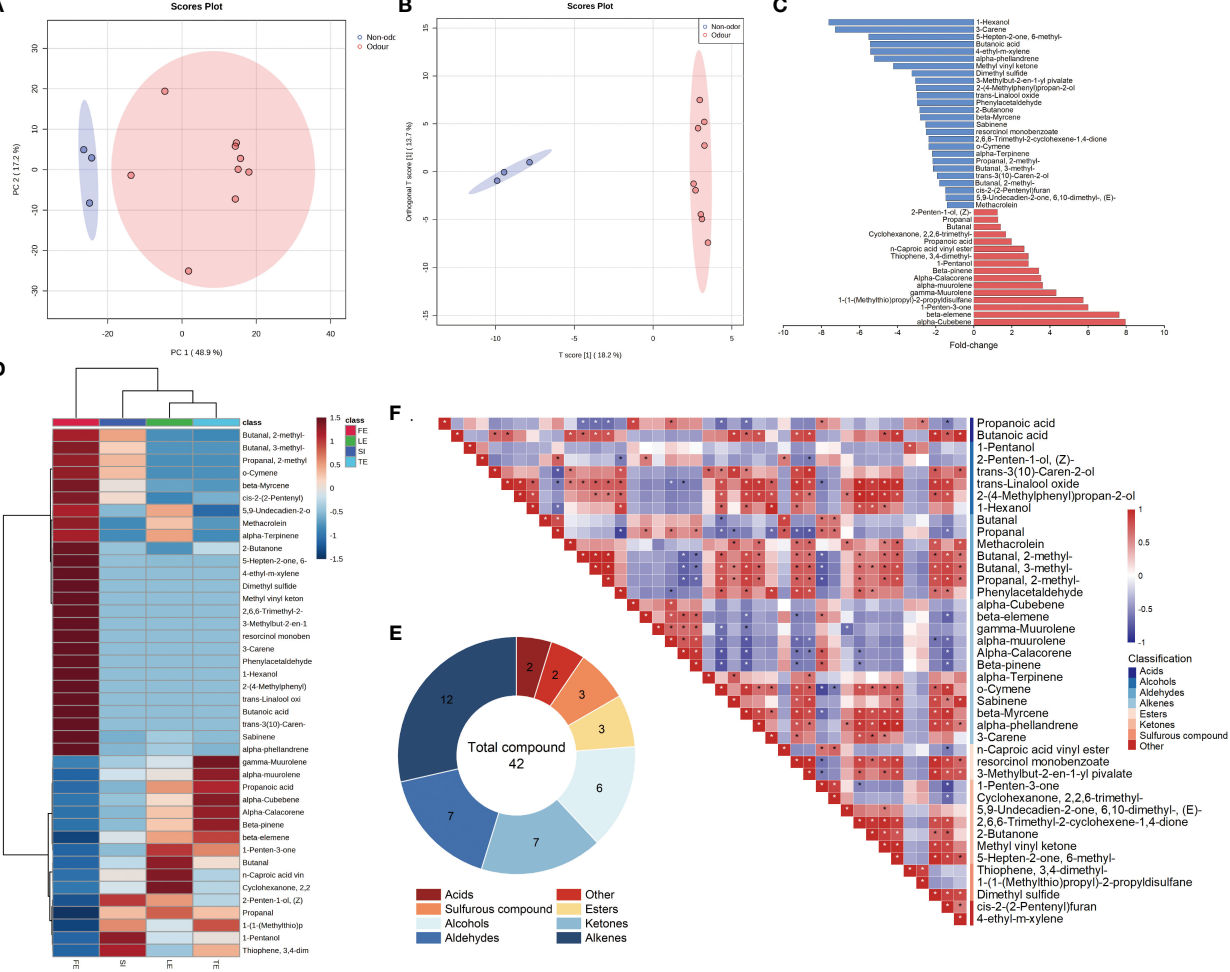
Multivariate statistical analysis of volatile organic compounds. **(A)** Principal component analysis of all samples. **(B)** OPLS-DA score plot of all samples. **(C)** Volcano plot of differential metabolites. **(D)** Cluster heat map of differential volatile metabolite. **(E)** Taxonomic donut plot of differential volatiles. **(F)** Correlation analysis diagram of differential volatiles. * indicates a significant correlation.

Multivariate analysis was performed based on the relative content of 166 VOCs using PCA and OPLS-DA to distinguish the four species of *Ferula*. Unsupervised PCA was used to evaluate the clustering of the four groups of *Ferula* samples. As shown in **Figure 2A**, the first principal component (PC) 1 and PC (PC2) accounted for 48.9% and 17.2% of the variation, respectively, with a cumulative contribution rate of 66.1%. *Ferula* samples from the different odour groups were all within the 95% confidence interval, indicating that the results were credible. As shown in the OPLS-DA plot (**Figure 2B**), samples from the four species of *Ferula* were divided into two groups based on whether they were edible or not, indicating significant differences in the metabolites. Further analysis of the odour characteristic compounds between the three strongly aromatic edible and nonedible (*F. feurlaeoides*) *Ferula* species was conducted to explore the composition differences; a correlation analysis was also performed.

To identify potential odour markers to distinguish the four species of *Ferula*, VIP and fold-change results from the OPLS-DA model were used to screen for the most representative metabolites. A total of 42 potential VOCs were screened; these metabolites could distinguish between the two different odours of *Ferula* leaf samples (**Figure 2C**). **Figures 2D, E** show an overview of the distribution of four characteristic metabolites of *Ferula*, including twelve alkenes, seven aldehydes, seven ketones, six alcohols, three esters, two acids, two sulphur-containing compounds, and three other classes. Twelve volatile metabolites were specific to *F. feurlaeoides*, while eight were specific to the three edible *Ferula* species.

Sixteen compounds showed an upregulated trend, representing a total of eight compound types (**Figure 2C**), mainly alkenes (eight species). Among them, eight compounds were common to the three edible *Ferula* species with special flavours; 1-Hexanol and β-elemene showed the highest upregulation trend. Most upregulated compounds were characteristic metabolites of edible *Ferula* with a pungent flavour. Although there were common compounds among the four *Ferula* species, the content in *F. feurlaeoides* was much lower than that in the other three species; therefore, these upregulated compounds are characteristic markers of *Ferula* consumption.

Twenty-six compounds were downregulated compounds, nine of which were different and12 of which were unique to *F. feurlaeoides*. In contrast to the upregulated compounds, the *F. feurlaeoides* content was advantageous. Dimethyl sulphide was unique to *F. feurlaeoides.* Although the content was extremely low, it was in line with our previous results showing that sulphide is a common constituent of *Ferula*. These downregulated compounds, mostly having fresh grassy and floral-fruity smell, are characteristic marker compounds of *F. feurlaeoides*.

Correlation analysis showed that butanoic acid, a significantly downregulated compound, was significantly positively correlated with dimethyl sulphide (*p* < 0.05) (**Figure 2F**). There was a negative correlation between compounds shared by the other three edible *Ferula* species, which was significant for compounds such as alcohols and ketones. In addition, two sulphides, thiophene,3,4-dimethyl- and 1-(1-(methylthio) propyl)-2-propyldisulfane, were significantly positively correlated with propanoic acid,1-pentano, propanoic acid (*p* < 0.05). These four compounds had a pungent odour and are important flavour sources for the three edible *Ferula* species. Among the alkene compounds, the specific compounds of edible *Ferula* exhibited an opposite trend to the alkene compounds of *F. feurlaeoides*. In general, the correlation between the compounds with a special flavour and the characteristic compounds of *F. feurlaeoides* showed an opposite trend, consistent with the results of the up- and downregulated compounds; therefore, the 42 compounds can represent the differential metabolites of these four species.

### Analysis of the composition of the root metabolites of four species of *Ferula*

**Figure 3:**
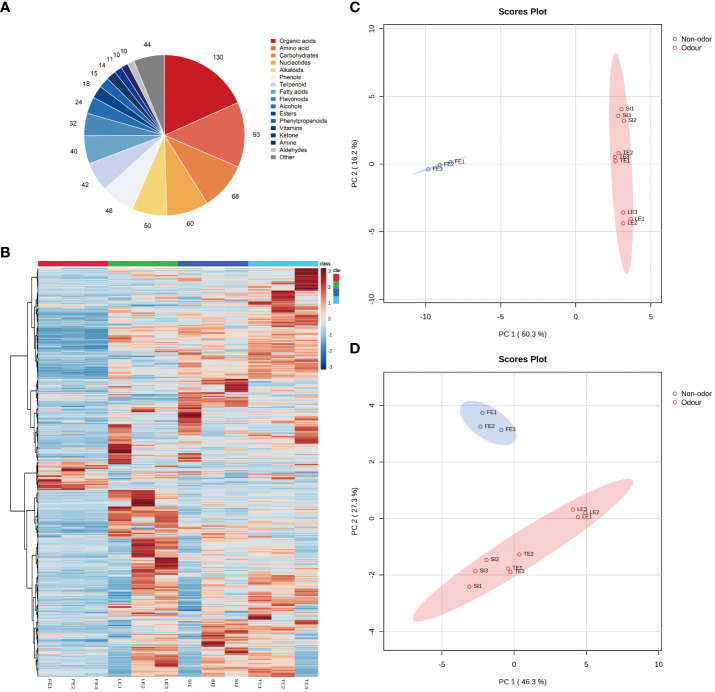
Metabolite analysis of *Ferula* root. **(A)** Pie chart of root metabolites. **(B)** heatmap of root metabolites. **(C)** PCA score plots in the positive mode. **(D)** PCA score plots in the negative ion mode.

The root samples of the four species of edible and nonedible *Ferula* were examined using non-targeted metabolomics; the overall relative content and species distribution are shown in **Figure 3A**. A total of 709 non-volatile metabolites of 17 classes were identified, which included 130 organic acids, 93 amino acids, 68 carbohydrates, 60 nucleotides, 50 alkaloids, 48 phenols, 42 terpenoids, 40 fatty acids, 32 flavonoids, 24 alcohols, 18 esters, 15 phenylpropanoids, 14 vitamins, 11 ketones, 10 amines, 10 aldehydes, and 87 other compounds.

Heatmaps based on all the identified metabolites showed that their variation and accumulation differed significantly across species (**Figure 3B**). The metabolite accumulation in the three species of edible *Ferula*, *i.e.*, *F. lehmannii*, *F. sinkiangensis*, and *F. teterrima*, was similar, and there was a clear distinction between them and the nonedible *F. feurlaeoides*. MetaboAnalyst was used for multivariate analysis of the data matrix of non-targeted metabolites from the root using Pareto correlation to differentiate between different groups of samples based on different metabolites. PCA showed that the metabolites were significantly different between the two groups. Data from the positive- and negative ion model samples confirmed their repeatability and reliability (**Figures 3C, D**). Further, results showed that the first two principal components contributed 76.5% of the cumulative contribution in the positive ion model (**Figure 2C**) (PC1 = 60.3%; PC2 = 16.2%). The total contribution in the negative ion model was 73.6% (PC1 = 46.3%, PC2 = 27.3%) (**Figure 2D**), and the contribution of both positive and negative ion models exceeded 70%, indicating that the model was reliable and that the good intergroup separation model was consistent with the results of the heat map.

### Screening of differential metabolites

**Figure 4:**
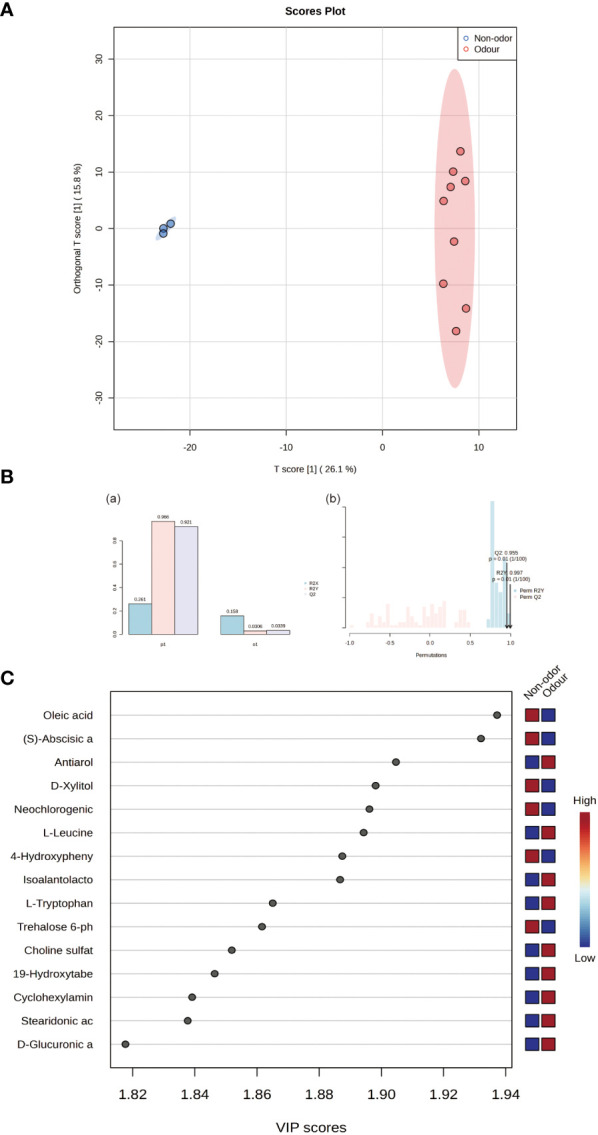
**(A)** OPLS-DA score plot of all samples. **(B)** Inertia histogram of model overview and 100 iterations of random permutation test of the OPLS-DA model. **(C)** Important features identified by OPLS-DA and VIP scores.

**Figure 4A** shows the OPLS-DA results. Edible and nonedible *Ferula* species were well separated in the model. To evaluate the performance of the OPLS-DA model, the goodness of fit (*R^2^*) and goodness of prediction (*Q^2^*) were selected as the statistical validation parameters.*R^2^* > 0.5 and *Q^2^* value close to 1.0 indicated a high degree of fi. In the OPLS-DA mode, the accuracies of *R^2^* and *Q^2^* were both > 0.9,further indicating the reliability of the model (**Figure 4B**).

**Figure 5:**
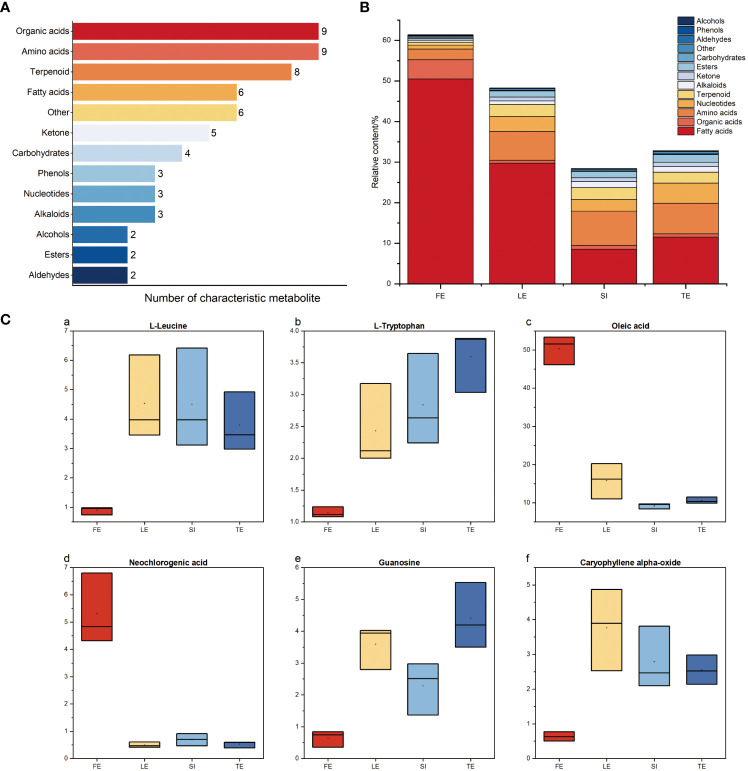
Differential metabolite profiles of edible and nonedible *Ferula*. **(A)** Number and type of differential metabolite. **(B)** Percentage of differential metabolites. **(C)** Distribution of key metabolites substances in the four *Ferula* (content>1%).

To further explore and screen the potential differential metabolites of the four species of *Ferula*, VIP score was calculated to assess the strength of the influence and explanatory power of each variable factor on the classification and differentiation of each group of samples (**Figure 4C**). The higher the VIP value, the greater the difference in metabolites between the groups and the more important it was to distinguish and classify the types of key bioactive components of *Ferula* (Scavarda et al., 2021). A total of 62 different metabolites were identified with VIP > 1, *p <*0.05 (*t*-test), and FC > 1 as thresholds (**Table 2**). As shown in **Figure 5A**, the 62 metabolites comprised 13 types, the most abundant of which were organic acids, amino acids, terpenoids, and fatty acids. **Figure 5B** shows the concentrations of the characteristic metabolites in the roots of the four species of *Ferula*. The fatty acid content was the highest; the fatty acid content of *F. feurlaeoides* accounted for more than half (50.48%) of all metabolites, while the organic acid content was much higher than that of the other three species of *Ferula*. However, the amino acid, terpene, nucleotide, alkaloid, and ester compound contents were much lower than those in the three edible *Ferula* species. For these characteristic metabolites, we selected those with content > 1% which included leucine, tryptophan, oleic acid, neochlorogenic acid, guanosine, and caryophyllene α oxide, for comparative analysis (**Figure 5C**).

**Table 2:** Differential metabolites of VOCs in four species of *Ferulas*.

Name	Fold Change	log^2^ (FC)	Class	Average relative content/%				Odor Description
				TE	LE	SI	FE	
α-Cubebene	248.79	7.96	Sesquiterpene hydrocarbons	0 ± 0 c	0.53 ± 0.16 ab	0.26 ± 0.14 ab	1.1 ± 0.58 a	vanilla, wax
β-elemene	196.66	7.62	`Sesquiterpene hydrocarbons	0 ± 0 a	0.32 ± 0.19 a	0.22 ± 0.11 a	0.4 ± 0.1 a	Herbal, waxy, fresh
1-Penten-3-one	63.93	6.00	Ketones	0 ± 0 c	2.08 ± 0.63 a	0.61 ± 0.55 bc	1.7 ± 0.01 ab	Spicy, garlic, mustard
1-(1-(Methylthio)propyl)-2-propyldisulfane	53.58	5.74	Sulfurous compound	0 ± 0 b	0.08 ± 0.05 b	0.06 ± 0.04 b	0.24 ± 0.02 a	sulfurous
γ-Muurolene	20.06	4.33	Sesquiterpene hydrocarbons	0 ± 0 c	0.1 ± 0.03 b	0.09 ± 0.04 bc	0.21 ± 0.02 a	herbal woody spice
α-muurolene	12.19	3.61	sesquiterpene hydrocarbons	0 ± 0 c	0.12 ± 0.03 ab	0.05 ± 0.03 bc	0.2 ± 0.04 a	Herbal, woody
α-Calacorene	11.45	3.52	Sesquiterpene hydrocarbons	0 ± 0 a	0.02 ± 0.01 a	0.05 ± 0.03 a	0.02 ± 0.01 a	woody
β-pinene	10.63	3.41	Monoterpene hydrocarbons	0 ± 0 a	0 ± 0 a	0.01 ± 0 a	0.01 ± 0 a	woody, pine, green, resinous
1-Pentanol	7.25	2.86	Alcohols	0.76 ± 0.36 c	7.73 ± 2.8 ab	3.52 ± 0.4 bc	13.12 ± 2.35 a	
Thiophene, 3,4-dimethyl-	7.23	2.85	Sulfurous compound	0.2 ± 0.09 a	7.37 ± 3.37 a	11.79 ± 8.78 a	13.41 ± 4 a	
*n*-Caproic acid vinyl ester	6.22	2.64	Esters	0.22 ± 0.13 b	2.3 ± 0.75 a	1.14 ± 0.36 ab	0.83 ± 0.52 ab	
Propanoic acid	3.94	1.98	Acids	0.01 ± 0 b	0.03 ± 0 ab	0.01 ± 0.01 ab	0.04 ± 0.01 a	Sour, spicy, fatty
Cyclohexanone, 2,2,6-trimethyl-	3.21	1.68	Ketones	0.02 ± 0.01 b	0.1 ± 0.02 a	0.05 ± 0.02 ab	0.04 ± 0.01 ab	grass, fragrant, flowers
Butanal	2.65	1.41	Aldehydes	0.01 ± 0 a	0.04 ± 0.01 a	0.02 ± 0.01 a	0.02 ± 0 a	pungent, cocoa, musty, green
Propanal	2.39	1.26	Aldehydes	0.08 ± 0.04 b	0.22 ± 0.04 a	0.19 ± 0.02 a	0.19 ± 0.02 a	
2-Penten-1-ol, (Z)-	2.36	1.24	Alcohols	0.98 ± 0.28 b	2.4 ± 0.69 ab	2.78 ± 0.63 a	1.75 ± 0.29 ab	
Methacrolein	0.38	-1.40	Aldehydes	0.02 ± 0.01 a	0.01 ± 0.01 a	0.01 ± 0 a	0.01 ± 0 a	
5,9-Undecadien-2-one, 6,10-dimethyl-, (*E*)-	0.36	-1.48	Ketones	0.12 ± 0.04 a	0.08 ± 0.06 a	0.03 ± 0.03 a	0 ± 0 a	
cis-2-(2-Pentenyl)furan	0.35	-1.50	Other	0.1 ± 0.02 a	0.02 ± 0.01 c	0.06 ± 0 b	0.03 ± 0.01 bc	
Butanal, 2-methyl-	0.28	-1.82	Aldehydes	0.16 ± 0.05 a	0.01 ± 0.01 b	0.11 ± 0.05 ab	0.01 ± 0.01 b	sweet, slightly, fruity
*trans*-3(10)-Caren-2-ol	0.26	-1.94	Alcohols	0.05 ± 0.02 a	0 ± 0 b	0 ± 0 b	0 ± 0 b	
Butanal, 3-methyl-	0.23	-2.13	Aldehydes	0.35 ± 0.1 a	0.03 ± 0.01 b	0.18 ± 0.1 ab	0.03 ± 0.01 b	unripe banana, apple flavor
Propanal, 2-methyl-	0.22	-2.16	Aldehydes	0.05 ± 0.01 a	0 ± 0 b	0.03 ± 0.01 ab	0 ± 0 b	fresh, aldehydic, floral, green
α-Terpinene	0.22	-2.18	Monoterpene hydrocarbons	0.08 ± 0.04 a	0.05 ± 0.05 a	0 ± 0 a	0 ± 0 a	
o-Cymene	0.19	-2.37	Monoterpene hydrocarbons	32.3 ± 1.99 a	0.4 ± 0.2 c	17.67 ± 3 b	0.68 ± 0.02 c	aromatic, smell
2,6,6-Trimethyl-2-cyclohexene-1,4-dione	0.19	-2.37	Ketones	0.02 ± 0.01 a	0 ± 0 b	0 ± 0 b	0 ± 0 b	musty, woody, sweet, tea, leaf
resorcinol monobenzoate	0.18	-2.51	Esters	0 ± 0 a	0 ± 0 b	0 ± 0 b	0 ± 0 b	
Sabinene	0.17	-2.53	Monoterpene hydrocarbons	10.38 ± 5.86 a	2.26 ± 0.9 a	1.39 ± 0.75 a	1.75 ± 0.24 a	
β-Myrcene	0.14	-2.82	Monoterpene hydrocarbons	12.73 ± 1.77 a	0.63 ± 0.3 c	4.66 ± 1.46 b	0.11 ± 0.09 c	light, balsam, aroma
2-Butanone	0.14	-2.85	Ketones	0.03 ± 0.01 a	0 ± 0 b	0 ± 0 b	0.01 ± 0 b	
Phenylacetaldehyde	0.13	-2.97	Aldehydes	0.7 ± 0.21 a	0 ± 0 b	0 ± 0 b	0 ± 0 b	flower, fruit, pollen, grass
trans-Linalool oxide	0.13	-2.99	Alcohols	0.05 ± 0.01 a	0 ± 0 b	0 ± 0 b	0 ± 0 b	floral, scent
2-(4-Methylphenyl)propan-2-ol	0.12	-3.02	Alcohols	0.1 ± 0.04 a	0 ± 0 b	0 ± 0 b	0 ± 0 b	
3-Methylbut-2-en-1-yl pivalate	0.12	-3.07	Esters	0.02 ± 0 a	0 ± 0 b	0 ± 0 b	0 ± 0 b	
Dimethyl sulfide	0.10	-3.26	Sulfurous compound	0.01 ± 0 a	0 ± 0 b	0 ± 0 b	0 ± 0 b	sulfurous
Methyl vinyl ketone	0.05	-4.23	Ketones	0.03 ± 0.01 a	0 ± 0 b	0 ± 0 b	0 ± 0 b	
α-phellandrene	0.03	-5.23	Monoterpene hydrocarbons	2.96 ± 1.51 a	0.23 ± 0.14 b	0 ± 0 b	0 ± 0 b	
4-ethyl-m-xylene	0.02	-5.42	Other	0.23 ± 0.21 a	0 ± 0 a	0 ± 0 a	0 ± 0 a	
Butanoic acid	0.02	-5.45	Acids	0.01 ± 0.01 a	0 ± 0 a	0 ± 0 a	0 ± 0 a	
5-Hepten-2-one, 6-methyl-	0.02	-5.52	Ketones	1.88 ± 0.69 a	0.03 ± 0.01 b	0.06 ± 0.01 b	0.03 ± 0.01 b	
3-Carene	0.01	-7.28	Monoterpene hydrocarbons	0.08 ± 0.04 a	0 ± 0 b	0 ± 0 b	0 ± 0 b	
1-Hexanol	0.01	-7.62	Alcohols	1.27 ± 0.62 a	0 ± 0 b	0 ± 0 b	0 ± 0 b	

Mean ± SE, n = 3. Different letters indicate significant species differences at the 0.05 level.

### Metabolic pathway analysis and interaction studies

**Figure 6:**
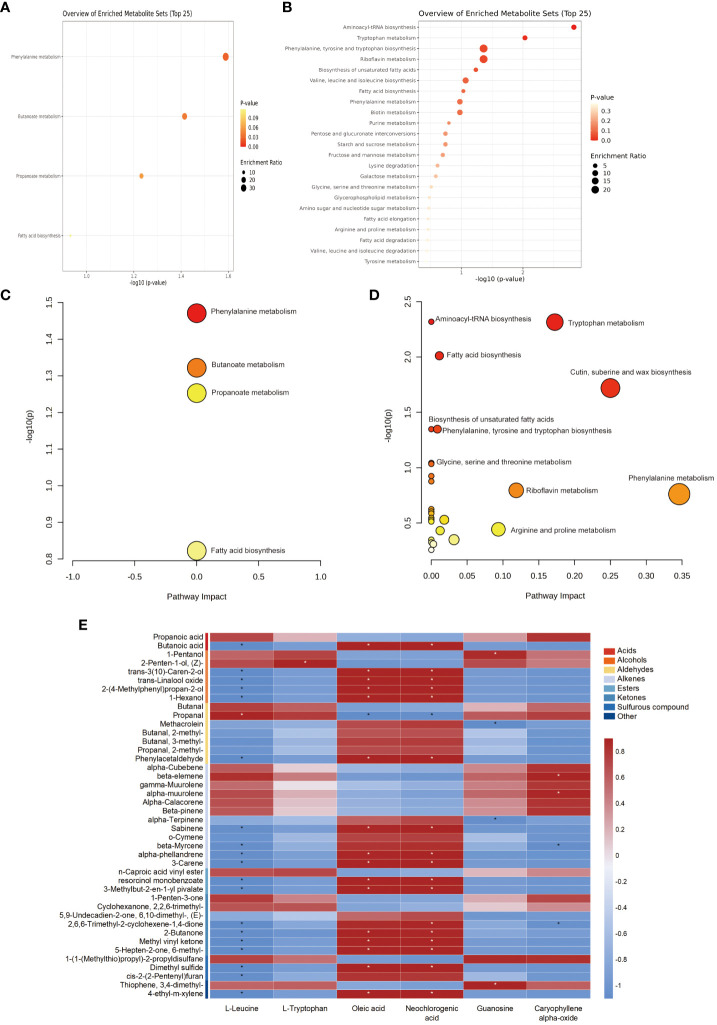
KEGG enrichment of the differential metabolites. **(A)** Volatile differential metabolites. **(B)** Root differential metabolites. **(C)** KEGG metabolic pathway classification of the differential metabolites in Leaf volatiles. **(D)** Root pharmacodynamic metabolites of KEGG metabolic pathway classification. **(E)** Correlation analysis among roots and volatile compounds in key characteristic metabolites of edible and nonedible *Ferula*. * indicates a significant correlation.

To further understand the metabolic pathways enriched in the differential metabolites, MetaboAnalyst was used to perform KEGG enrichment analysis of the selected differential metabolites of edible and nonedible *Ferula* species. Here, the 42 and 62 significantly different metabolites identified in the volatile and bioactive characteristic metabolites of *Ferula*, respectively, were distributed across 30 metabolic pathways (**Figures 6A, B**). The eight most significant metabolic pathways were phenylalanine metabolism; butanoate metabolism of VOC differential metabolites; aminoacyl-tRNA biosynthesis; tryptophan metabolism; fatty acid biosynthesis; cutin, suberin, and wax biosynthesis; biosynthesis of unsaturated fatty acids; and biosynthesis of phenylalanine, tyrosine, and tryptophan. Fatty acid biosynthesis and phenylalanine metabolism pathways were enriched in both volatile characteristic metabolites and bioactive differential metabolites of *Ferula* (**Figures 6C, D**).

Subsequently, we correlated the volatile differential metabolites with six major characteristic metabolites of the bioactive components and found consistent correlation trends between the two typical amino acid metabolites (**Figure 6E**). The characteristic volatiles of *F. feurlaeoides* showed a significant negative correlation (*p* < 0.05), and the characteristic volatiles of the three edible *Ferula* species were positively correlated. Since most aroma components of *Ferula* are ketones, alcohols, acids, and esters, a large part of the aroma precursors synthesised by these compounds originate from amino acids. Among the bioactive components, nucleotides were significantly positively correlated with guanosine and 1-pentanol (*p <*0.05) and with sulphide 1-[1-(methylthio)propyl]-2-propyldisulfane. Conversely, methacrolein and α-terpinene levels showed a significant negative correlation (*p* < 0.05).

Oleic and neochlorogenic acids are the characteristic bioactive components of *F. feurlaeoides*, and their contents are higher than those in edible *Ferula*. This study showed that oleic and neochlorogenic acids were positively correlated with aromatic volatiles, such as alcohols, phenylacetaldehyde, alkenes, and ketones. Pungent or foul-smelling compounds, such as propanoic acid, 1-pentanol, butanal, propanal, 1-penten-3-one, 1-[1-(methylthio)propyl]-2-propyldisulfane, and thiophene,3,4-dimethyl-, showed a negative correlation. In fatty acid metabolism, unsaturated fatty acids are important precursors for the formation of volatile aroma compounds, which also explains the positive correlation between predominant fatty acid compounds in *F. feurlaeoides* and various aroma compounds. Terpenoid compounds, such as caryophyllene α-oxide, were significantly positively correlated with β-elemene and α-muurolene, two alkenes with fresh grass and herbal flavour (*p* < 0.05). There was a significant negative correlation between β-myrcene and 2,6, 6-trimethyl-2-cyclohexene-1,4-dione (*p* < 0.05).

## Discussion

Changes in aroma composition influence aroma quality (Zheng et al., 2022). The flavour of *Ferula* is derived from the odour produced by the various volatile compounds. Therefore, volatile compound content influences the aroma characteristics of different species of *Ferula*. Differences in flavour among different types of Ferula were initially investigated. The combination of solid phase microextraction and SPME-GC-MS has the characteristics of high sensitivity, high accuracy and rapid operation, and is a common means to study volatile aroma compounds in food. In this study, HS-SPME/GC-MS was used to detect the volatile components of four species of *Ferula*. The ketone compound 1-penten-3-one, which is common among the three edible *Ferula* species, has a pungent onion-like taste (Li et al., 2017). Sulphur-containing substances generally have a low threshold value and strong odour, presenting a pungent taste similar to that of garlic, onion, and leek. According to the National Standards of the People’s Republic of China, Classification of natural spices (GB/T 21725-2017), *Ferula* belongs to the spice-type of plants, which is supported by this study. The compounds (*Z*)-sec-Butyl propenyl disulphide and (*E*)-sec-butyl propenyl disulfide are present in both *F. teterrima* and *F. sinkiangensis*, with concentrations exceeding 1%. They are also detected in *F. lehmannii*, but not in the non-edible *F. feurlaeoides*. Previous studies have shown that these sulphur compounds are representative compounds of *Ferula* (Muturi et al., 2018; Boghrati and Iranshahi, 2019; Sonigra and Meena, 2021) as also been shown that volatile sulphur compounds can be used to determine the type and origin of *Ferula* (Volatile sulphur compounds: The possible metabolite pattern to identify the sources and types of asafoetida by headspace GC/MS analysis). However, although *F. feurlaeoides* contains sulphide, the amount is too low to be a major contributor to the flavour. The presence of sulphur imparts a unique flavor to *Ferula*, thus making it one of the significant distinguishing factors between edible and nonedible *Ferula* in this study.

A number of studies have shown that terpene compounds are an important part of the volatile oils and volatiles of *Ferula* (Salehi et al., 2019). Among the 42 potential VOCs screened, monoterpene hydrocarbons and sesquiterpene hydrocarbons have the highest content and the most abundant species. β-pinene, o-Cymene, Sabinene and β-Myrcene are all compounds found in *Ferula*, accounting for more than 80% of the total alkene compounds. Among them, β-pinene had the highest content among the monoterpene hydrocarbons in the three edible *Ferula* species, while the other three monoterpene hydrocarbons had the highest content in *F. feurlaeoides.* These compounds are also highly reported representative compounds of *Ferula*. Studies have shown that terpene compounds mostly have floral, citrus, and woody aroma (Quan et al., 2023), which contributes to the aroma of *Ferula*. Alcohols and aldehydes are mostly fresh and grassy compounds, while compounds unique to *F. feurlaeoides* mostly have floral and fruity aroma. Compounds, such as 1-hexanol and 5-hepten-2-one, 6-methyl, have a sweet citrus odour; these fresh-smelling compounds are significantly more abundant in *F. feurlaeoides* than in the remaining three species. Therefore, compared with the non-edible *F. feurlaeoides*, which has a higher proportion of alkene, the many sulphide and ketone compounds in edible species contribute to the rich flavor of *Ferula.*

As a traditional medicinal part, the roots of *Ferula* contain significant chemical composition groups. UHPLC-MS/MS offers faster analysis, improved resolution, and higher sensitivity compared to HPLC-MS/MS when used in conjunction with tandem mass spectrometric detection (Rathod et al., 2019). Therefore, we performed metabolite profiling of the roots of edible and non-edible *Ferula* species by untargeted UHPLC-MS/MS. After multivariate analysis, we identified 62 metabolites that contributed the most to the flavour of the four species main constituents of *Ferula*. Subsequently, we selected six metabolites as primary characteristic components, including fatty acids, amino acids, organic acids, nucleotides, and terpenoids. These metabolites contribute significantly to the overall flavor and medicinal value of *Ferula*. Relevant research indicates that *Ferula*, among numerous spice species within the Apiaceae family, exhibits a higher content of fatty acids. Oleic acid, in particular, emerges as a significant constituent of the fatty acid composition in *Ferula* (Daga et al., 2022). The oleic acid content was high in all four species of *Ferula*; however, the oleic acid content of *F. feurlaeoides* was significantly higher than that of the other three species of *Ferula*. As a typical monounsaturated fatty acid, oleic acid is easily digested and absorbed by the human body (Zhou et al., 2019). The four species of *Ferula* included in this study were medicinal plants, and the high fatty acid content reflects the value of *Ferula* as a traditional medicinal plant. Neochlorogenic acid, a phenolic acid, is an isomer of chlorogenic acids with various pharmacological activities, such as antioxidant, anti-spasmodic, and antiviral effects; it promotes liver glucose utilisation and inhibits carcinogenic mutagenesis. Neochlorogenic acid is used in the pharmaceutical and healthcare industries, chemical industries, and functional food industries (Mehmood et al., 2022). Neochlorogenic acid and oleic acid are important characteristic compounds of *F. feurlaeoides* and the most abundant fatty acids and organic acids.

Free amino acids are important flavour-forming water-soluble compounds (Pei et al., 2014). The four *Ferula* species were rich in free amino acid compounds. L-leucine and L-tryptophan were the two major amino acid metabolites. Among them, L-tryptophan was bitter, and the amino acid content of edible *Ferula* was significantly higher than that of *F. feurlaeoides* (*p* < 0.05), indicating that tryptophan significantly contributes to the overall flavour. Furthermore, amino acids, which serve as the cornerstone of a healthy diet, have also been found in abundance in *F. assa-foetida L*. (Singh et al., 2023). Guanosine is also an important characteristic metabolite in the four species of *Ferula*. Nucleosides are important metabolites involved in regulatory processes, protection against myocardial damage, and antiviral activity (Du et al., 2015). Natural nucleosides are not only used as biomarkers for the diagnosis of human diseases but also as important markers for the detection of bioactive ingredients during the quality evaluation of Chinese medicinal materials. In addition, nucleotides can have a synergistic effect with amino acids to enhance food flavour (Zhao et al., 2020).

In this study, the roots of the four species of *Ferula* contained large amounts of olefins such as caryophyllene α-oxide. Moreover, olefins were observed to be abundant in various *Ferula* species such as *F. tunetana* Pomel ex Batt (Baccari et al., 2023). Caryophyllene α-oxide, a well-known oxygenated sesquiterpene, exhibits a wide range of protective effects in pharmaceutical, nutritional, and cosmetic applications. Additionally, it possesses various pharmacological and biological activities, including anti-inflammatory, antifungal, analgesic, and anticholinesterase properties (Jassal et al., 2021).These terpenoids exhibit strong antibacterial and anticarcinogenic activities along with antioxidant, anti-asthma, anti-anxiety, and analgesic activities (Chen et al., 2021). *Ferula lehmannii*, *F. sinkiangensis*, and *F. teterrima* not only have edible value but are also more valuable than *F. feurlaeoides*. Indeed, their medicinal composition is much higher than that of *F. feurlaeoides*, consistent with traditional cognition. Therefore, the large number of rich amino acids, nucleotides, and alkenes contribute to the good nutritional value and unique flavour of *Ferula*. These results indicate that the six key metabolites selected in this study have good medicinal value and are representative, consistent with our previous findings (Jiang et al., 2022; Jiang et al., 2023).

According to the characteristic difference in VOCs from the *Ferula* species acid and the main active components of *Ferula*, the pathways related to the flavor and medicinal value of different types of *Ferula* were further studied. We performed KEGG metabolic pathway analysis of the major metabolites of *Ferula*. The amino acid pathway is the main pathway for the synthesis of flavour-characteristic volatile compounds in the leaves and roots of *Ferula*. Previous studies have shown that changes in phenylalanine content are positively correlated with aromatic substances. Phenylalanine is a source of phenolic metabolites. In this study, the common characteristic metabolite of the bioactive components, phenylacetaldehyde, was also a key metabolite of phenylalanine metabolism through acylation, methylation, hydroxylation, and other reactions. Ultimately, phenylacetaldehyde affects the synthesis of volatile compounds, such as phenylacetaldehyde in volatiles (Aragüez and Valpuesta Fernández, 2013). Phenylacetaldehyde is a volatile compound abundant in to *F. feurlaeoides*. Tyrosine, tryptophan, and phenylalanine are involved in protein synthesis and are precursors of many natural products, such as alkaloids and cell wall components. Tryptophan is a precursor of several secondary metabolites such as glucosinolates and alkaloids, which enhance the biosynthesis of aromatic compounds (Miao et al., 2022). In addition to their bioactive composition, the volatiles of edible and nonedible *Ferula* species differ based on the type of organic acid; however, in terms of bioactive composition, nonedible *F. feurlaeoides* and edible *Ferula* have similar biosynthetic pathways and produce similar metabolites. The difference lies in the types of volatile odours and their bioactive content.

Fatty acids are important metabolites in the biosynthesis of aromatic volatile compounds (Zhou et al., 2019). In the fatty acid metabolism pathway, hydroperoxides generated from unsaturated fatty acids catalysed by lipoxygenase are converted into aromatic compounds by hydroperoxidase (Yang et al., 2022). In this study, the fatty acid content of the four species of *Ferula* was the highest among the various metabolites. Fatty acids were the key metabolite products of *Ferula*. Five fatty acids were identified in the enrichment pathway; however, 1-hexanol involved in fatty acid biosynthesis was a unique volatile of *F. feurlaeoides*. These differences in metabolites suggest that the difference between edible and nonedible *Ferula* also lies in fatty acid content and type. Previous studies have reported that there is a significant synergistic effect between umami amino acids and flavoured nucleotides and that the formation of sulphur-containing compounds is affected by enzymatic reactions with amino acids as precursors (Yu et al., 2020). Common amino acid metabolism aroma precursors, such as L-Leucine and L-Tryptophan, and flavour compounds were obtained via a two-step reaction of transamination and decarboxylation (Jia et al., 2022). Therefore, amino acids, nucleotides, and terpenoids play key roles in VOC formation from different species of *Ferula*.

## Conclusion

This study found that the unique odour of edible *Ferula* was attributed to the presence of volatile compounds, such as acids, aldehydes, alkenes, and sulphur-containing compounds. In addition, the bioactive components of the three edible *Ferula* species were significantly associated with pungent odours, whereas those of the nonedible *F. feurlaeoides* were positively associated with fresh fruity and grassy aromas and negatively associated with pungent volatiles. This answers our second research question that the smell of *Ferula* is linked to the bioactive ingredients, and the stronger the odour, the higher the content of major bioactive ingredients, such as amino acids and nucleotides. Odour profiling can be used for screening and assessing *Ferula* quality. The understanding of odour-bioactivity relationship opens doors for novel food, cosmetic, and medicinal product development. In summary, our results demonstrate the relationship between differential metabolites and provides a valuable foundation and potential avenues for further exploration.

## Data availability statement

The raw/processed data required to reproduce these findings cannot be shared at this time as the data also forms part of an ongoing study, but are available from the corresponding author on reasonable request. Requests to access the datasets should be directed to Li Zhuang, zhuanglishzu@foxmail.com.

## Author contributions

MJ: Writing – original draft, Writing – review & editing, Conceptualization, Data curation, Formal analysis, Investigation, Methodology, Project administration, Software, Visualization. MP: Writing – review & editing. YL: Writing – review & editing. GL: Writing – review & editing. XL: Writing – review & editing. LZ: Funding acquisition, Project administration, Writing – review & editing, Resources.

## Supplementary material

Supplementary Figure 1 Global distribution of *Ferula* species. Global distribution information for the genus Ferula was obtained from the Global Biodiversity Information Facility Database (http://www.gbif.org/), and mapped using the ArcGIS 10.8 software.
