# Supplementary figures and images for: Quality evaluation of four Ferula plants and identification of their key volatiles based on non-targeted metabolomics
Source: Front Plant Sci. 2024 Jan 4;14:1297449. doi: 10.3389/fpls.2023.1297449 (PMC10794503; doi:10.3389/fpls.2023.1297449)

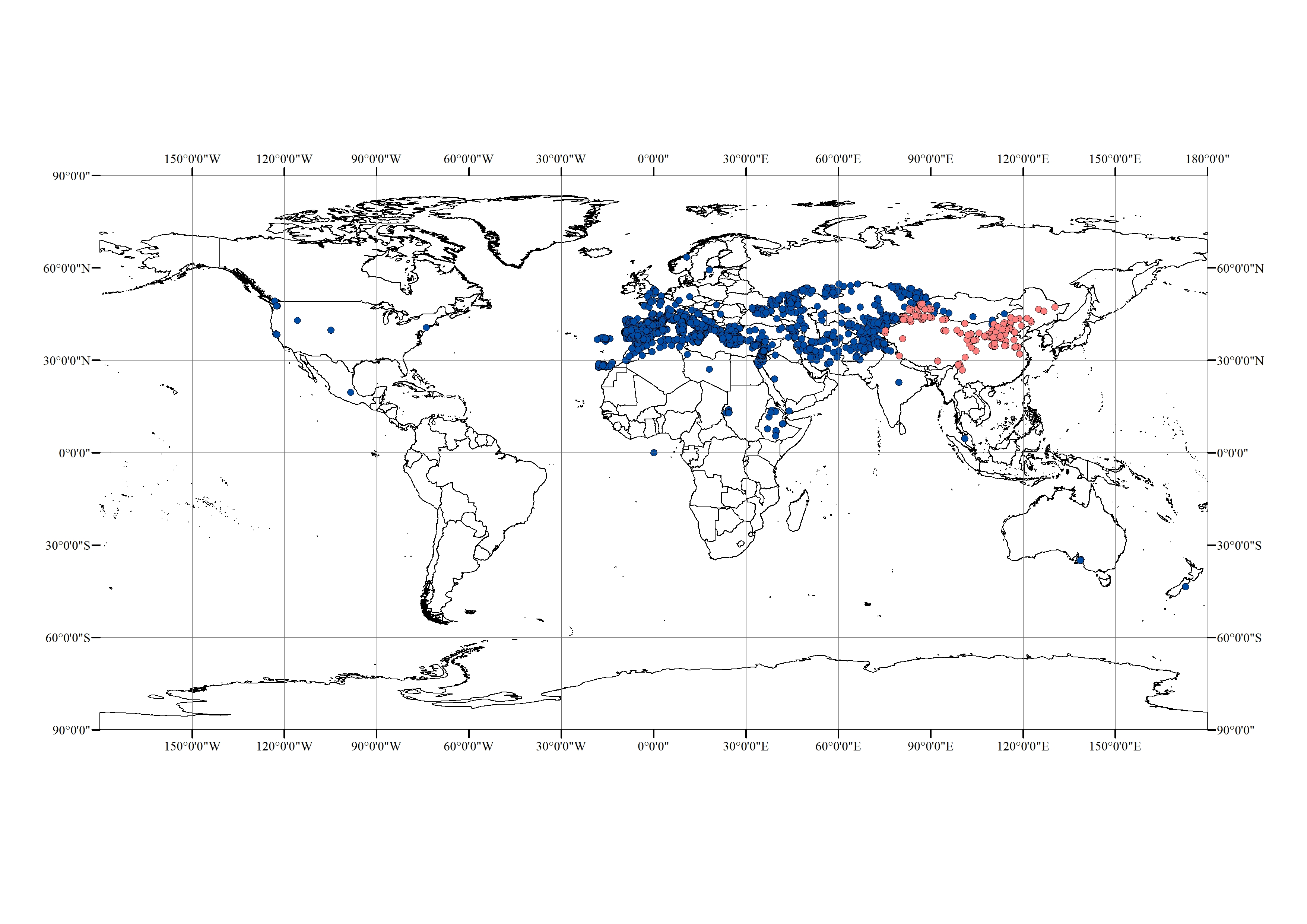

Supplement: Supplementary Figure 1 — Global distribution of Ferula species. Global distribution information for the genus Ferula was obtained from the Global Biodiversity Information Facility Database (http://www.gbif.org/), and mapped using the ArcGIS 10.8 software. [file Image_1.jpg]
