# Supplementary material for: Quality evaluation of four Ferula plants and identification of their key volatiles based on non-targeted metabolomics
Source: Front Plant Sci. 2024 Jan 4;14:1297449. doi: 10.3389/fpls.2023.1297449 (PMC10794503; doi:10.3389/fpls.2023.1297449)
Supplement: Supplementary file 2 [file Table_1.docx]

| **Supplementary Table 1 Experiment materials** | | | | |
| --- | --- | --- | --- | --- |
| Species | Coordinate information | Collection locality | Voucher number | Specimen storage location |
| *F.* *sinkiangensis* | 44°13′N, 86°02′E | Shihezi City, Xinjiang | JM2022020101 | SHI |
| *F. teterrima* | 44°13′N, 86°02′E | Shihezi City, Xinjiang | JM2022020201 | SHI |
| *F. lehmannii* | 44°13′N, 86°02′E | Shihezi City, Xinjiang | JM2022020301 | SHI |
| *F. feurlaeoides* | 44°13′N, 86°02′E | Shihezi City, Xinjiang | JM2022020401 | SHI |
