# Supplementary material for: Quality evaluation of four Ferula plants and identification of their key volatiles based on non-targeted metabolomics
Source: Front Plant Sci. 2024 Jan 4;14:1297449. doi: 10.3389/fpls.2023.1297449 (PMC10794503; doi:10.3389/fpls.2023.1297449)
Supplement: Supplementary file 3 [file Table_2.docx]

| **Table A.1 Identification of VOCs in four species of *Ferula*** | | | | | | | | | | | | | | |  |
| --- | --- | --- | --- | --- | --- | --- | --- | --- | --- | --- | --- | --- | --- | --- | --- |
| Name | CAS | | rt/s | Formula | MZ | Class | Average relative content/% | | | | | | | |  |
|  |  | |  |  |  |  | TE | | LE | | SI | | FE | |  |
| Ethanol | | 64-17-5 | 198.782 | C2H6O | 46.07 | Alcohols | | 0±0 | | 0.035±0.025 | | 0.248±0.152 | | 0.081±0.07 | |
| Methanethiol | | 74-93-1 | 102.815 | CH4S | 48.1075 | Alcohols | | 0.003±0.002 | | 0.001±0.001 | | 0.002±0.002 | | 0±0 | |
| 2-Propenal | | 107-02-8 | 143.498 | C3H4O | 56.063 | Aldehydes | | 0.012±0.008 | | 0.02±0.008 | | 0.018±0.004 | | 0.022±0.007 | |
| Propanal | | 123-38-6 | 123.155 | C3H6O | 58.079 | Aldehydes | | 0.189±0.019 | | 0.22±0.039 | | 0.191±0.019 | | 0.077±0.04 | |
| Acetic acid | | 64-19-7 | 1073.765 | C2H4O2 | 60.052 | Acids | | 0.06±0.026 | | 0.03±0.03 | | 0.016±0.016 | | 0±0 | |
| Acetaldehyde, hydroxy- | | 141-46-8 | 171.270 | C2H4O2 | 60.052 | Aldehydes | | 0.925±0.041 | | 1.505±0.073 | | 2.835±0.172 | | 0.964±0.301 | |
| Dimethyl sulfide | | 75-18-3 | 113.121 | C2H6S | 62.134 | Sulfurous compound | | 0±0 | | 0±0 | | 0±0 | | 0.008±0.002 | |
| 1,4-Pentadiene | | 591-93-5 | 102.045 | C5H8 | 68.117 | Alkenes | | 0.006±0.004 | | 0.002±0.001 | | 0.01±0.007 | | 0.008±0.004 | |
| Methyl vinyl ketone | | 78-94-4 | 208.293 | C4H6O | 70.0898 | Ketones | | 0±0 | | 0±0 | | 0±0 | | 0.034±0.013 | |
| Methacrolein | | 78-85-3 | 159.398 | C4H6O | 70.09 | Aldehydes | | 0.006±0.003 | | 0.014±0.006 | | 0.005±0.003 | | 0.022±0.008 | |
| 2-Butenal | | 4170-30-3 | 337.372 | C4H6O | 70.09 | Aldehydes | | 0.143±0.048 | | 0.116±0.041 | | 0.043±0.027 | | 0.04±0.027 | |
| Formic acid, ethenyl ester | | 692-45-5 | 105.683 | C3H4O2 | 72.0627 | Esters | | 0.075±0.013 | | 0.098±0.024 | | 0.107±0.043 | | 0.07±0.016 | |
| 2-Butanone | | 78-93-3 | 170.613 | C4H8O | 72.1057 | Ketones | | 0.007±0.004 | | 0±0 | | 0.004±0.002 | | 0.03±0.013 | |
| Propanal, 2-methyl- | | 78-84-2 | 129.406 | C4H8O | 72.106 | Aldehydes | | 0±0 | | 0±0 | | 0.028±0.013 | | 0.048±0.014 | |
| Butanal | | 123-72-8 | 156.284 | C4H8O | 72.106 | Aldehydes | | 0.024±0.004 | | 0.037±0.008 | | 0.02±0.012 | | 0.01±0.004 | |
| Propanoic acid | | 79-09-4 | 1208.710 | C3H6O2 | 74.078 | Acids | | 0.035±0.013 | | 0.028±0.004 | | 0.013±0.005 | | 0.006±0.004 | |
| Acetic acid, methyl ester | | 79-20-9 | 135.148 | C3H6O2 | 74.078 | Esters | | 0±0 | | 0.002±0 | | 0.038±0.012 | | 0.001±0 | |
| Carbamic acid, monoammonium salt | | 1111-78-0 | 89.793 | CH6N2O2 | 78.07 | Acids | | 0.718±0.279 | | 0.989±0.392 | | 0.839±0.351 | | 1.077±0.371 | |
| Furan, 2-methyl- | | 534-22-5 | 165.796 | C5H6O | 82.101 | Other | | 0.003±0.002 | | 0.006±0.006 | | 0.011±0.004 | | 0.009±0.005 | |
| 2-Pentenal, (*E*)- | | 1576-87-0 | 498.397 | C5H8O | 84.116 | Aldehydes | | 0.084±0.047 | | 0.108±0.035 | | 0.191±0.115 | | 0.177±0.07 | |
| 1-Penten-3-one | | 1629-58-9 | 299.429 | C5H8O | 84.116 | Ketones | | 1.701±0.01 | | 2.077±0.631 | | 0.608±0.551 | | 0±0 | |
| Butanal, 2-methyl- | | 96-17-3 | 178.435 | C5H10O | 86.13 | Aldehydes | | 0.011±0.006 | | 0.015±0.009 | | 0.11±0.054 | | 0.162±0.049 | |
| Pentanal | | 110-62-3 | 240.770 | C5H10O | 86.132 | Aldehydes | | 0.057±0.007 | | 0.124±0.032 | | 0.091±0.048 | | 0.073±0.023 | |
| Butanal, 3-methyl- | | 590-86-3 | 181.825 | C5H10O | 86.132 | Aldehydes | | 0.029±0.006 | | 0.029±0.006 | | 0.179±0.099 | | 0.348±0.096 | |
| 3-Pentanone | | 96-22-0 | 238.683 | C5H10O | 86.132 | Ketones | | 0.231±0.063 | | 0.212±0.074 | | 0.359±0.158 | | 0.365±0.057 | |
| 1-Penten-3-ol | | 616-25-1 | 568.285 | C5H10O | 86.132 | Alcohols | | 0.611±0.585 | | 0.036±0.019 | | 1.106±1.037 | | 1.324±0.398 | |
| 2-Penten-1-ol, (*Z*)- | | 1576-95-0 | 861.644 | C5H10O | 86.1323 | Alcohols | | 1.749±0.295 | | 2.402±0.691 | | 2.781±0.631 | | 0.981±0.285 | |
| Butanoic acid | | 107-92-6 | 1486.940 | C4H8O2 | 88.105 | Acids | | 0±0 | | 0±0 | | 0±0 | | 0.012±0.007 | |
| 1-Pentanol | | 71-41-0 | 736.475 | C5H12O | 88.148 | Alcohols | | 0.025±0.012 | | 0.021±0.012 | | 0.049±0.026 | | 0±0 | |
| Dimethyl sulfone | | 67-71-0 | 382.877 | C2H6O2S | 94.13 | Sulfurous compound | | 0.002±0.001 | | 0±0 | | 0.01±0.005 | | 0±0 | |
| Furan, 2-ethyl- | | 3208-16-0 | 212.086 | C6H8O | 96.127 | Other | | 2.655±0.456 | | 4.236±1.879 | | 1.116±0.985 | | 1.444±0.605 | |
| 2,4-Hexadienal, (E,E)- | | 142-83-6 | 996.839 | C6H8O | 96.127 | Aldehydes | | 1.873±0.927 | | 1.942±1.609 | | 0.617±0.572 | | 0.201±0.189 | |
| 2-Hexenal | | 505-57-7 | 672.328 | C6H10O | 98.143 | Aldehydes | | 16.901±2.723 | | 14.009±6.287 | | 14.865±9.051 | | 7.757±2.437 | |
| 3-Hexenal | | 4440-65-7 | 515.091 | C6H10O | 98.143 | Aldehydes | | 0.02±0.007 | | 0.134±0.066 | | 0.033±0.022 | | 0.049±0.039 | |
| 2-Pentenal, 2-methyl- | | 623-36-9 | 1309.610 | C6H10O | 98.143 | Aldehydes | | 0±0 | | 0.011±0.006 | | 0.017±0.01 | | 0±0 | |
| 2,3-Pentanedione | | 600-14-6 | 373.753 | C5H8O2 | 100.116 | Ketones | | 0.049±0.008 | | 0.032±0.008 | | 0.042±0.024 | | 0.036±0.01 | |
| Cubenene | | 29837-12-5 | 1535.450 | C8H4 | 100.117 | Alkenes | | 0.527±0.193 | | 0.067±0.053 | | 0.11±0.059 | | 0±0 | |
| Hexanal | | 66-25-1 | 406.088 | C6H12O | 100.159 | Aldehydes | | 5.748±0.612 | | 14.154±3.692 | | 0.039±0.003 | | 2.02±0.897 | |
| Butanoic acid, 2-methyl- | | 116-53-0 | 1394.890 | C5H10O2 | 102.132 | Acids | | 0.013±0.003 | | 0±0 | | 0.003±0.003 | | 0±0 | |
| Pentanoic acid | | 109-52-4 | 1486.420 | C5H10O2 | 102.132 | Acids | | 0.001±0.001 | | 0.006±0.003 | | 0.004±0.002 | | 0±0 | |
| 1-Hexanol | | 111-27-3 | 919.007 | C6H14O | 102.175 | Alcohols | | 0±0 | | 0±0 | | 0±0 | | 1.269±0.62 | |
| Benzaldehyde | | 100-52-7 | 1181.070 | C7H6O | 106.122 | Aldehydes | | 0.219±0.039 | | 0.859±0.28 | | 0.708±0.105 | | 0.532±0.168 | |
| 2,4-Heptadienal, (E,E)- | | 4313-03-5 | 1139.600 | C7H10O | 110.154 | Aldehydes | | 0.967±0.127 | | 1.433±0.299 | | 1.495±0.343 | | 1.151±0.555 | |
| Dihydro-3-methylene-5-methyl-2-furanone | | 62873-16-9 | 1431.145 | C6H8O2 | 112.127 | Esters | | 0.174±0.03 | | 0.129±0.059 | | 0.155±0.044 | | 0.113±0.032 | |
| 2-Heptenal, (*E*)- | | 18829-55-5 | 859.681 | C7H12O | 112.17 | Aldehydes | | 0.028±0.02 | | 0.082±0.041 | | 0.139±0.046 | | 0.067±0.024 | |
| 1-Hepten-3-one | | 2918-13-0 | 821.084 | C7H12O | 112.17 | Ketones | | 0.02±0.01 | | 0.005±0.005 | | 0.01±0.008 | | 0.008±0.008 | |
| 4-Heptenal, (*Z*)- | | 6728-31-0 | 710.916 | C7H12O | 112.17 | Aldehydes | | 0.016±0.002 | | 0±0 | | 0.003±0.003 | | 0±0 | |
| Thiophene, 3,4-dimethyl- | | 632-15-5 | 725.781 | C6H8S | 112.193 | Sulfurous compound | | 0.006±0.003 | | 0.003±0.001 | | 0.008±0.005 | | 0±0 | |
| Hexanoic acid | | 142-62-1 | 1626.060 | C6H12O2 | 116.158 | Acids | | 0±0 | | 0.087±0.066 | | 0.006±0.006 | | 0.059±0.045 | |
| Hydroxymethyl 2-hydroxy-2-methylpropionate | | 2110-78-3 | 627.477 | C5H10O3 | 118.131 | Esters | | 0.007±0.002 | | 0.005±0.003 | | 0.03±0.019 | | 0.006±0.003 | |
| Phenylacetaldehyde | | 122-78-1 | 1355.135 | C8H8O | 120.148 | Aldehydes | | 0±0 | | 0±0 | | 0±0 | | 0.705±0.206 | |
| (*E*)-1-Methyl-2-(prop-1-en-1-yl)disulfane | | 23838-19-9 | 788.880 | C4H8S2 | 120.236 | Sulfurous compound | | 0.006±0.003 | | 0.001±0.001 | | 0.047±0.035 | | 0±0 | |
| 2,5-Furandicarboxaldehyde | | 823-82-5 | 1243.920 | C6H4O3 | 124.094 | Other | | 0.012±0.006 | | 0.053±0.01 | | 0.042±0.023 | | 0.018±0.015 | |
| 3,5-Octadien-2-one, (E,E)- | | 30086-02-3 | 1179.770 | C8H12O | 124.18 | Ketones | | 0.05±0.05 | | 0.071±0.071 | | 0.072±0.038 | | 0.061±0.013 | |
| 1,4-Hexadiene, 3,3,5-trimethyl- | | 74753-00-7 | 1251.995 | C9H16 | 124.223 | Alkenes | | 0.128±0.064 | | 0.304±0.218 | | 0.066±0.066 | | 0.015±0.013 | |
| 2,6,6-Trimethyl-2-cyclohexene-1,4-dione | | 1125-21-9 | 1422.280 | C9H12O2 | 126.15 | Ketones | | 0±0 | | 0±0 | | 0.005±0.005 | | 0.018±0.006 | |
| 5-Hepten-2-one, 6-methyl- | | 110-93-0 | 884.331 | C8H14O | 126.196 | Ketones | | 0.03±0.007 | | 0.033±0.01 | | 0.06±0.007 | | 1.88±0.689 | |
| 3,3-Diethoxy-1-propyne | | 10160-87-9 | 457.191 | C7H12O2 | 128.169 | Aldehydes | | 0.007±0.003 | | 0.002±0.002 | | 0.006±0.006 | | 0±0 | |
| Octanal | | 124-13-0 | 799.309 | C8H16O | 128.212 | Aldehydes | | 0.031±0.02 | | 0.011±0.002 | | 0.009±0.005 | | 0.021±0.006 | |
| Propanoic acid, anhydride | | 123-62-6 | 572.184 | C6H10O3 | 130.142 | Acids | | 0.352±0.352 | | 0.587±0.336 | | 0.019±0.014 | | 0±0 | |
| Benzaldehyde, 3-ethyl- | | 34246-54-3 | 1444.430 | C9H10O | 134.175 | Aldehydes | | 0.075±0.071 | | 0.029±0.013 | | 0.018±0.002 | | 0.03±0.016 | |
| o-Cymene | | 527-84-4 | 756.542 | C10H14 | 134.218 | Alkenes | | 0.684±0.023 | | 0.395±0.202 | | 17.673±2.997 | | 32.297±1.985 | |
| 1,3,8-p-Menthatriene | | 18368-95-1 | 964.898 | C10H14 | 134.218 | Alkenes | | 0.047±0.027 | | 0±0 | | 0±0 | | 0.024±0.013 | |
| Benzene, tert-butyl- | | 98-06-6 | 901.781 | C10H14 | 134.218 | Alkenes | | 0±0 | | 0.015±0.011 | | 0.005±0.005 | | 0.007±0.004 | |
| cosmene | | 460-01-5 | 1050.940 | C10H14 | 134.218 | Alkenes | | 0.091±0.013 | | 0.01±0.005 | | 0.006±0.006 | | 0±0 | |
| 4-ethyl-m-xylene | | 874-41-9 | 1247.040 | C10H14 | 134.218 | Other | | 0±0 | | 0±0 | | 0±0 | | 0.225±0.212 | |
| cis-2-(2-Pentenyl)furan | | 70424-13-4 | 818.859 | C9H12O | 136.191 | Other | | 0.031±0.006 | | 0.019±0.01 | | 0.057±0.001 | | 0.103±0.015 | |
| (1R)-2,6,6-Trimethylbicyclo[3.1.1]hept-2-ene | | 7785-70-8 | 288.490 | C10H16 | 136.23 | Alkenes | | 1.787±0.205 | | 1.123±0.478 | | 1.082±0.694 | | 2.436±0.515 | |
| alpha-thujene | | 2867-05-2 | 299.957 | C10H16 | 136.234 | Alkenes | | 0.069±0.04 | | 0.145±0.125 | | 0.002±0.002 | | 0.197±0.162 | |
| Camphene | | 79-92-5 | 359.417 | C10H16 | 136.234 | Alkenes | | 0.199±0.012 | | 0.931±0.406 | | 0.292±0.203 | | 0.334±0.068 | |
| Beta-pinene | | 18172-67-3 | 425.909 | C10H16 | 136.234 | Alkenes | | 13.124±2.347 | | 7.728±2.795 | | 3.521±0.396 | | 0.764±0.359 | |
| Sabinene | | 3387-41-5 | 466.056 | C10H16 | 136.234 | Alkenes | | 1.748±0.243 | | 2.257±0.898 | | 1.386±0.753 | | 10.378±5.864 | |
| Cyclohexene, 4-methylene-1-(1-methylethyl)- | | 99-84-3 | 473.095 | C10H16 | 136.234 | Alkenes | | 0±0 | | 0±0 | | 0.025±0.016 | | 0±0 | |
| 3-Carene | | 13466-78-9 | 518.213 | C10H16 | 136.234 | Alkenes | | 0±0 | | 0±0 | | 0±0 | | 0.082±0.04 | |
| alpha-phellandrene | | 99-83-2 | 549.758 | C10H16 | 136.234 | Alkenes | | 0±0 | | 0.23±0.14 | | 0.005±0.002 | | 2.957±1.507 | |
| beta-Myrcene | | 123-35-3 | 566.327 | C10H16 | 136.234 | Alkenes | | 0.115±0.09 | | 0.631±0.3 | | 4.659±1.458 | | 12.732±1.772 | |
| D-Limonene | | 5989-27-5 | 623.562 | C10H16 | 136.234 | Alkenes | | 9.558±2.5 | | 11.192±5.917 | | 4.847±2.808 | | 5.497±4.075 | |
| Bicyclo[3.1.0]hex-2-ene, 4-methyl-1-(1-methylethyl)- | | 28634-89-1 | 635.031 | C10H16 | 136.234 | Alkenes | | 0.97±0.541 | | 0.058±0.058 | | 0.519±0.401 | | 0.092±0.089 | |
| *trans-beta-Ocimene* | | 3779-61-1 | 701.012 | C10H16 | 136.234 | Alkenes | | 0.141±0.052 | | 0.063±0.048 | | 0.014±0.009 | | 0.036±0.022 | |
| gamma-Terpinene | | 99-85-4 | 710.657 | C10H16 | 136.234 | Alkenes | | 0.044±0.005 | | 0.833±0.537 | | 9.512±4.859 | | 6.281±6.09 | |
| Terpinolene | | 586-62-9 | 776.636 | C10H16 | 136.234 | Alkenes | | 4.366±0.992 | | 0±0 | | 0±0 | | 0.048±0.046 | |
| 2,4,6-Octatriene, 2,6-dimethyl- | | 673-84-7 | 977.146 | C10H16 | 136.234 | Alkenes | | 0.083±0.075 | | 0.048±0.048 | | 0.101±0.101 | | 0±0 | |
| alpha-Terpinene | | 99-86-5 | 583.398 | C10H16 | 136.234 | Alkenes | | 0±0 | | 0.055±0.047 | | 0±0 | | 0.083±0.042 | |
| Tricyclo[2.2.1.0(2,6)]heptane, 1,7,7-trimethyl- | | 508-32-7 | 266.311 | C10H16 | 136.234 | Ketones | | 0.033±0.033 | | 0.434±0.148 | | 0.036±0.036 | | 0±0 | |
| Furan, 2-pentyl- | | 3777-69-3 | 691.622 | C9H14O | 138.207 | Other | | 0.048±0.027 | | 0.245±0.085 | | 0.249±0.023 | | 0.179±0.046 | |
| 2-Cyclohexen-1-one, 4-(1-methylethyl)- | | 500-02-7 | 1388.900 | C9H14O | 138.207 | Ketones | | 0±0 | | 0.321±0.167 | | 0.008±0.005 | | 0.045±0.004 | |
| 1H-Pyrrole-2,5-dione, 3-ethyl-4-methyl- | | 20189-42-8 | 2111.720 | C7H9NO2 | 139.152 | Ketones | | 0.015±0.004 | | 0.028±0.009 | | 0.013±0.002 | | 0.018±0.006 | |
| 2-Nonenal, (E)- | | 18829-56-6 | 1201.660 | C9H16O | 140.223 | Alkenes | | 0.007±0.007 | | 0.015±0.009 | | 0.016±0.008 | | 0.016±0.008 | |
| Cyclohexanone, 2,2,6-trimethyl- | | 2408-37-9 | 834.781 | C9H16O | 140.223 | Ketones | | 0.041±0.008 | | 0.096±0.023 | | 0.046±0.021 | | 0.019±0.005 | |
| 3-Hexen-1-ol, acetate, (*Z*)- | | 3681-71-8 | 850.426 | C8H14O2 | 142.196 | Esters | | 0.024±0.024 | | 0.03±0.024 | | 0.002±0.002 | | 0±0 | |
| *n-Caproic* acid vinyl ester | | 3050-69-9 | 868.149 | C8H14O2 | 142.196 | Esters | | 0.832±0.52 | | 2.3±0.749 | | 1.136±0.356 | | 0.216±0.132 | |
| Nonanal | | 124-19-6 | 979.743 | C9H18O | 142.24 | Aldehydes | | 0.042±0.021 | | 0.026±0.007 | | 0.015±0.007 | | 0.017±0.003 | |
| 2-Furanmethanol, tetrahydro-, acetate | | 637-64-9 | 1665.830 | C7H12O3 | 144.168 | Esters | | 0.045±0.023 | | 0.031±0.018 | | 0.024±0.024 | | 0.033±0.011 | |
| 3-Methyl-4-isopropylphenol | | 3228-02-2 | 2058.005 | C10H14O | 150.218 | Other | | 0±0 | | 0.02±0.02 | | 0±0 | | 0.008±0.002 | |
| Furan, 3-(4-methyl-3-pentenyl)- | | 539-52-6 | 1019.665 | C10H14O | 150.218 | Alkenes | | 0.24±0.01 | | 2.144±0.961 | | 0.16±0.115 | | 0±0 | |
| 2-(4-Methylphenyl)propan-2-ol | | 1197-01-9 | 1633.225 | C10H14O | 150.218 | Alcohols | | 0±0 | | 0±0 | | 0±0 | | 0.095±0.036 | |
| Pinocarvone | | 30460-92-5 | 1238.970 | C10H14O | 150.218 | Ketones | | 0.01±0.01 | | 0.035±0.027 | | 0.022±0.022 | | 0.005±0.003 | |
| Dehydrocampfer | | 22516-10-5 | 331.501 | C10H14O | 150.218 | Ketones | | 0.021±0.002 | | 0.014±0.011 | | 0±0 | | 0±0 | |
| (*E*)-4,8-Dimethylnona-1,3,7-triene | | 19945-61-0 | 1220.970 | C11H18 | 150.261 | Alkenes | | 0.449±0.053 | | 2.679±1.345 | | 0.227±0.115 | | 0±0 | |
| 2-Cyclohexen-1-one, 3-methyl-6-(1-methylethyl)- | | 89-81-6 | 1466.610 | C10H16O | 152.23 | Ketones | | 0.025±0.018 | | 0.048±0.039 | | 0±0 | | 0.026±0.01 | |
| *trans-3*(10)-Caren-2-ol | | - | 1162.300 | C10H16O | 152.23 | Alcohols | | 0±0 | | 0±0 | | 0±0 | | 0.049±0.024 | |
| 1-Cyclohexene-1-carboxaldehyde, 2,6,6-trimethyl- | | 432-25-7 | 1318.105 | C10H16O | 152.233 | Aldehydes | | 0.121±0.025 | | 0.167±0.064 | | 0.162±0.02 | | 0.092±0.02 | |
| 3-Butene-1,2-diol, 1-(2-furanyl)- | | 19261-13-3 | 1307.530 | C8H10O3 | 154.163 | Alcohols | | 0.003±0.001 | | 0±0 | | 0±0 | | 0.001±0.001 | |
| Linalool | | 78-70-6 | 1227.360 | C10H18O | 154.25 | Alkenes | | 0.021±0.01 | | 0.415±0.297 | | 0.009±0.009 | | 0.119±0.041 | |
| Nonane, 3,7-dimethyl- | | 17302-32-8 | 905.576 | C11H24 | 156.308 | Other | | 0.02±0.015 | | 0.016±0.009 | | 0.004±0.002 | | 0.012±0.007 | |
| 4-Hexen-1-ol, acetate | | 72237-36-6 | 848.867 | C8H16O3 | 160.211 | Acids | | 0.006±0.006 | | 0.005±0.004 | | 0.016±0.016 | | 0±0 | |
| (*Z*)-sec-Butyl propenyl disulfide | | 24351-70-0 | 1085.370 | C7H14S2 | 162.316 | Sulfurous compound | | 1.057±1.057 | | 0.042±0.019 | | 2.748±1.379 | | 0±0 | |
| (*E*)-sec-Butyl propenyl disulfide | | 24351-71-1 | 1101.010 | C7H14S2 | 162.316 | Sulfurous compound | | 2.597±1.586 | | 0.144±0.134 | | 1.62±0.799 | | 0±0 | |
| Benzene, 2-methoxy-4-methyl-1-(1-methylethyl)- | | 1076-56-8 | 1284.840 | C11H16O | 164.244 | Alkenes | | 0±0 | | 0.009±0.009 | | 0.302±0.151 | | 0.025±0.015 | |
| Benzene, 1-methoxy-4-methyl-2-(1-methylethyl)- | | 31574-44-4 | 1300.755 | C11H16O | 164.244 | Other | | 0±0 | | 0.062±0.062 | | 0.271±0.204 | | 0.175±0.079 | |
| *n-Propyl* sec-butyl disulfide | | 59849-54-6 | 1003.355 | C7H16S2 | 164.332 | Sulfurous compound | | 0.411±0.193 | | 0.019±0.019 | | 0.236±0.219 | | 0±0 | |
| 1-(1-(Methylthio)propyl)-2-propyldisulfane | | 126876-22-0 | 1700.250 | C7H16S3 | 168.344 | Sulfurous compound | | 13.409±3.995 | | 7.374±3.37 | | 11.795±8.78 | | 0.203±0.088 | |
| Disulfide, methyl 1-(methylthio)propyl | | 53897-66-8 | 1700.900 | C5H12S3 | 168.344 | Sulfurous compound | | 0.019±0.019 | | 5.841±3.117 | | 3.858±3.844 | | 0.114±0.06 | |
| 3-Methylbut-2-en-1-yl pivalate | | 211429-71-9 | 984.059 | C10H18O2 | 170.2487 | Esters | | 0±0 | | 0±0 | | 0±0 | | 0.021±0.005 | |
| *trans-Linalool* oxide | | 34995-77-2 | 1078.075 | C10H18O2 | 170.249 | Alcohols | | 0±0 | | 0±0 | | 0±0 | | 0.051±0.011 | |
| 1,3-Dioxolane, 2-heptyl- | | 4359-57-3 | 1649.655 | C10H20O2 | 172.265 | Aldehydes | | 0.01±0.009 | | 0±0 | | 0.002±0.002 | | 0.004±0.002 | |
| 1-Pentanone, 1-(4-methylphenyl)- | | 1671-77-8 | 1037.140 | C12H16O | 176.255 | Ketones | | 0.005±0.003 | | 0.012±0.012 | | 0.024±0.014 | | 0±0 | |
| Disulfide, bis(1-methylpropyl) | | 5943-30-6 | 1057.720 | C8H18S2 | 178.359 | Sulfurous compound | | 0.319±0.242 | | 0±0 | | 0.379±0.102 | | 0±0 | |
| 2(4H)-Benzofuranone, 5,6,7,7a-tetrahydro-4,4,7a-trimethyl-, (R)- | | 17092-92-1 | 2186.555 | C11H16O2 | 180.243 | Esters | | 0.01±0.001 | | 0.024±0.004 | | 0.008±0.005 | | 0.012±0.005 | |
| prenyl benzoate | | 5205-11-8 | 1886.690 | C12H14O2 | 190.238 | Esters | | 0±0 | | 0.016±0.012 | | 0.02±0.02 | | 0.003±0.001 | |
| Benzene, (1-methoxy-4-methyl-3-pentenyl)- | | 68705-86-2 | 947.695 | C13H18O | 190.28 | Alkenes | | 0±0 | | 0.001±0.001 | | 0±0 | | 0±0 | |
| *trans-beta-Ionone* | | 79-77-6 | 1737.800 | C13H20O | 192.297 | Ketones | | 0±0 | | 0.422±0.224 | | 0±0 | | 0.057±0.049 | |
| 5,9-Undecadien-2-one, 6,10-dimethyl-, (*E*)- | | 3796-70-1 | 1637.660 | C13H22O | 194.313 | Ketones | | 0±0 | | 0.084±0.064 | | 0.03±0.03 | | 0.121±0.039 | |
| 5,9-Undecadien-2-one, 6,10-dimethyl-, (*Z*)- | | 3879-26-3 | 1638.440 | C13H22O | 194.313 | Alkenes | | 0.016±0.008 | | 0.015±0.015 | | 0.018±0.013 | | 0±0 | |
| Alpha-Calacorene | | 21391-99-1 | 1707.280 | C15H20 | 200.319 | Alkenes | | 0.196±0.039 | | 0.117±0.031 | | 0.047±0.026 | | 0±0 | |
| Naphthalene, 1,2,3,4-tetrahydro-1,6-dimethyl-4-(1-methylethyl)-, (1S-cis)- | | 483-77-2 | 1602.590 | C15H22 | 202.335 | Alkenes | | 0.045±0.045 | | 0.255±0.234 | | 0.017±0.017 | | 0±0 | |
| alpha-muurolene | | 31983-22-9 | 1460.870 | C15H24 | 204 | Alkenes | | 0.207±0.025 | | 0.104±0.033 | | 0.086±0.044 | | 0±0 | |
| (+)-epi-Bicyclosesquiphellandrene | | 54274-73-6 | 1414.315 | C15H24 | 204.35 | Alkenes | | 0.082±0.047 | | 0.212±0.192 | | 0.006±0.006 | | 0±0 | |
| beta-ylangene | | 20479-06-5 | 1245.090 | C15H24 | 204.35 | Alkenes | | 0.061±0.009 | | 0.011±0.005 | | 0.002±0.002 | | 0±0 | |
| Copaene | | 3856-25-5 | 1124.750 | C15H24 | 204.351 | Alkenes | | 1.698±1.126 | | 0.18±0.1 | | 0.346±0.293 | | 0±0 | |
| beta-cubebene | | 13744-15-5 | 1197.230 | C15H24 | 204.351 | Alkenes | | 0.134±0.134 | | 0.075±0.02 | | 0.004±0.004 | | 0±0 | |
| Caryophyllene | | 87-44-5 | 1283.800 | C15H24 | 204.351 | Alkenes | | 0.171±0.068 | | 0±0 | | 0.098±0.084 | | 0±0 | |
| (-)-Aristolene | | 6831-16-9 | 1235.575 | C15H24 | 204.351 | Alkenes | | 0.455±0.105 | | 0.011±0.011 | | 0.098±0.096 | | 0±0 | |
| (*E*)-beta-Famesene | | 18794-84-8 | 1388.120 | C15H24 | 204.351 | Alkenes | | 0.185±0.112 | | 0.001±0.001 | | 0.053±0.053 | | 0.095±0.038 | |
| (Z,E)-alpha-Farnesene | | 26560-14-5 | 1474.680 | C15H24 | 204.351 | Alkenes | | 0.001±0.001 | | 0.02±0.017 | | 0±0 | | 0±0 | |
| Naphthalene, 1,2,3,5,6,8a-hexahydro-4,7-dimethyl-1-(1-methylethyl)-, (1S-cis)- | | 483-76-1 | 1503.890 | C15H24 | 204.351 | Alkenes | | 0.538±0.538 | | 0±0 | | 0.193±0.193 | | 0.093±0.046 | |
| beta-elemene | | 515-13-9 | 1275.210 | C15H24 | 204.351 | Alkenes | | 0.4±0.101 | | 0.318±0.194 | | 0.217±0.112 | | 0±0 | |
| alpha-Cubebene | | 17699-14-8 | 1075.460 | C15H24 | 204.351 | Alkenes | | 1.103±0.581 | | 0.534±0.165 | | 0.26±0.141 | | 0±0 | |
| (-)-beta-Bourbonene | | 5208-59-3 | 1163.475 | C15H24 | 204.351 | Alkenes | | 0.193±0.05 | | 0.002±0.002 | | 0.173±0.164 | | 0±0 | |
| bicyclogermacren | | 24703-35-3 | 1471.040 | C15H24 | 204.351 | Alkenes | | 0.555±0.555 | | 0.898±0.898 | | 0.475±0.07 | | 0±0 | |
| Germacrene D | | 23986-74-5 | 1437.535 | C15H24 | 204.351 | Alkenes | | 2.055±1.008 | | 0.077±0.039 | | 0.572±0.301 | | 0±0 | |
| gamma-Muurolene | | 30021-74-0 | 1410.030 | C15H24 | 204.351 | Alkenes | | 0.237±0.021 | | 0.082±0.054 | | 0.056±0.041 | | 0±0 | |
| Naphthalene, 1,2,4a,5,8,8a-hexahydro-4,7-dimethyl-1-(1-methylethyl)-, [1S-(1a,4ab,8aa)]- | | 523-47-7 | 1503.640 | C15H24 | 204.351 | Alkenes | | 0.619±0.291 | | 0.48±0.185 | | 0.049±0.049 | | 0±0 | |
| 1-Isopropyl-4,7-dimethyl-1,2,3,4,5,6-hexahydronaphthalene | | 16729-00-3 | 1370.255 | C15H24 | 204.351 | Other | | 0.128±0.071 | | 0.003±0.003 | | 0±0 | | 0±0 | |
| 1H-Cyclopropa[a]naphthalene, 1a,2,3,5,6,7,7a,7b-octahydro-1,1,7,7a-tetramethyl-, [1aR-(1a alpha,7alpha,7aalpha,7b alpha)]- | | 17334-55-3 | 1305.975 | C15H24 | 204.351 | Alkenes | | 0.312±0.301 | | 0.001±0.001 | | 0.011±0.011 | | 0±0 | |
| 1H-Cycloprop[e]azulene, 1a,2,3,5,6,7,7a,7b-octahydro-1,1,4,7-tetramethyl-, [1aR-(1a alpha,7alpha,7a beta,7b alpha)]- | | 21747-46-6 | 1419.935 | C15H24 | 204.351 | Alkenes | | 0.028±0.014 | | 0.088±0.046 | | 0±0 | | 0±0 | |
| 1,4,7,-Cycloundecatriene, 1,5,9,9-tetramethyl-, Z,Z,Z- | | - | 1381.980 | C15H24 | 204.3511 | Alkenes | | 0.266±0.066 | | 0.347±0.248 | | 0.085±0.085 | | 0±0 | |
| 6-Methyl-6-(5-methylfuran-2-yl)heptan-2-one | | 50464-95-4 | 1802.465 | C13H20O2 | 208.297 | Ketones | | 0.038±0.009 | | 0±0 | | 0.019±0.019 | | 0.011±0.006 | |
| Ethanone, 1-(4-hydroxyphenyl)-2-phenyl- | | 2491-32-9 | 2004.820 | C14H12O2 | 212.244 | Ketones | | 0±0 | | 0.003±0.003 | | 0.002±0.001 | | 0±0 | |
| resorcinol monobenzoate | | 136-36-7 | 1365.690 | C13H10O3 | 214.217 | Esters | | 0±0 | | 0±0 | | 0±0 | | 0.004±0 | |
| Disulfide, bis[1-(methylthio)ethyl] | | 69078-77-9 | 1603.495 | C6H14S4 | 214.435 | Sulfurous compound | | 0.02±0.008 | | 0±0 | | 0.084±0.047 | | 0±0 | |
| 3-Methylbut-2-enoic acid, 4-nitrophenyl ester | | - | 1271.945 | C11H11NO4 | 221.21 | Alkenes | | 0.011±0.011 | | 0.082±0.071 | | 0.008±0.008 | | 0±0 | |
| 2,6-Octadienal, 3,7-dimethyl-, (*E*)- | | 141-27-5 | 1479.640 | C10H16O | 224.339 | Aldehydes | | 0.07±0.004 | | 0.462±0.238 | | 0.077±0.02 | | 0.05±0.013 | |
| 13-Methyltetradecanal | | 75853-51-9 | 1662.430 | C15H30O | 226.398 | Other | | 0±0 | | 0.026±0.014 | | 0.023±0.012 | | 0±0 | |
| Hexadecane | | 544-76-3 | 827.738 | C16H34 | 226.441 | Alcohols | | 0.041±0.002 | | 0.041±0.021 | | 0.045±0.026 | | 0.022±0.011 | |
| 2-Hexanol, 3,3,5-trimethyl-2-(3-methylphenyl)- | | 274266-33-0 | 1632.960 | C16H26O | 234.38 | Alcohols | | 0.013±0.013 | | 0±0 | | 0.018±0.006 | | 0±0 | |
| 1-Phenyl-1-decanol | | 21078-95-5 | 1559.960 | C16H26O | 234.38 | Esters | | 0.015±0.015 | | 0±0 | | 0.004±0.003 | | 0±0 | |
| Butanoic acid, 2-methyl-, 3,7-dimethyl-2,6-octadienyl ester, (*E*)- | | 68705-63-5 | 1691.115 | C15H26O2 | 238.366 | Other | | 0.392±0.203 | | 0.094±0.045 | | 0.008±0.004 | | 0±0 | |
| Hexane, 3,4-bis(1,1-dimethylethyl)-2,2,5,5-tetramethyl- | | 62850-21-9 | 574.792 | C18H38 | 254.494 | Esters | | 0.159±0.159 | | 0.179±0.178 | | 0.002±0.002 | | 0.001±0.001 | |
| 1,2-Benzenedicarboxylic acid, bis(2-methylpropyl) ester | | 84-69-5 | 2388.665 | C16H22O4 | 278.344 | Alkenes | | 0.008±0.005 | | 0.004±0.004 | | 0.011±0.006 | | 0.006±0.003 | |
| Neophytadiene | | 504-96-1 | 1727.880 | C20H38 | 278.516 | Aldehydes | | 0.005±0.005 | | 0.01±0.004 | | 0.026±0.015 | | 0±0 | |
| 1,3-Dioxolane, 2-pentadecyl- | | 4360-57-0 | 1649.930 | C18H36O2 | 284.477 | Alkenes | | 0±0 | | 0±0 | | 0.004±0.003 | | 0.001±0.001 | |
| 1,2-Benzenediol, O-(2-furoyl)-O-(pentafluoropropionyl)- | | - | 1839.750 | C21H26O | 294.4 | Alkenes | | 0.01±0.01 | | 0.001±0.001 | | 0±0 | | 0±0 | |
| 1-Hexene, 3-methyl-6-phenyl-4-(1-phenylethoxy)- | | - | 971.800 | C21H26O | 294.4 | Alcohols | | 0.006±0.005 | | 0.001±0.001 | | 0±0 | | 0±0 | |
| Heptaethylene glycol | | 5617-32-3 | 2650.595 | C14H30O8 | 326.383 | Alcohols | | 0.007±0.001 | | 0±0 | | 0.001±0.001 | | 0±0 | |
| 1,3-Dithiane, 2,2-[(3,4-diphenyl-1,2-cyclobutanediyl)bis(methylene)]bis- | | 74744-59-5 | 1121.487 | C26H32S4 | 472.8 | Sulfurous compound | | 0±0 | | 0.002±0.002 | | 0±0 | | 0±0 | |
